# Supplementary material for: RadBone: bone toxicity following pelvic radiotherapy – a prospective randomised controlled feasibility study evaluating a musculoskeletal health package in women with gynaecological cancers undergoing pelvic radiotherapy
Source: BMJ Open. 2022 Jun 14;12(6):e056600. doi: 10.1136/bmjopen-2021-056600 (PMC9198686; doi:10.1136/bmjopen-2021-056600)
Supplement: Author's manuscript [file bmjopen-2021-056600.draft_revisions.pdf]

BMJ Open is committed to open peer review. As part of this commitment we make the peer review history of every article we publish publicly available.

When an article is published we post the peer reviewers' comments and the authors' responses online. We also post the versions of the paper that were used during peer review. These are the versions that the peer review comments apply to.

The versions of the paper that follow are the versions that were submitted during the peer review process. They are not the versions of record or the final published versions. They should not be cited or distributed as the published version of this manuscript.

BMJ Open is an open access journal and the full, final, typeset and author-corrected version of record of the manuscript is available on our site with no access controls, subscription charges or pay-per-view fees (http://bmjopen.bmj.com).

If you have any questions on BMJ Open's open peer review process please email info.bmjopen@bmj.com

BMJ Open

RadBone: Bone Toxicity following Pelvic Radiotherapy. A prospective randomised controlled feasibility study evaluating a musculoskeletal health package in women with gynaecological cancers undergoing pelvic radiotherapy.

Journal:	*BMJ Open*
Manuscript ID	bmjopen-2021-056600
Article Type:	Protocol
Date Submitted by the Author:	19-Aug-2021

Complete List of Authors:	Chatzimavridou Grigoriadou, Victoria; Christie Hospital NHS Foundation Trust, Department of Endocrinology; University of Manchester, Manchester Academic Health Science Centre<br>Barraclough, Lisa H; Christie Hospital NHS Foundation Trust, Department of Clinical Oncology<br>Baricevic-Jones, Ivona; Manchester Academic Health Science Centre, Stoller Biomarker Discovery Centre, Division of Cancer Sciences, School of Medical Sciences, Faculty of Biology, Medicine and Health, University of Manchester<br>Bristow, Robert; University of Manchester, Manchester Cancer Research Centre<br>Eden, Martin; The University of Manchester Manchester Centre for Health Economics<br>Haslett, Kate; Christie Hospital NHS Foundation Trust, Department of Clinical Oncology<br>Johnson, Karen; Christie Hospital NHS Foundation Trust, Department of Clinical Oncology<br>Kochhar, Rohit; The Christie National Health Service Foundation Trust, Department of Radiology<br>Merchant, Zoe; Programme Lead for the Greater Manchester 'Prehab4Cancer and Recovery programme'/Highly Specialist Occupational Therapist. GM Cancer alliance hosted by the Christie NHS Foundation Trust.<br>Moore, John; GM Cancer Clinical Director for Prehabilitation and Recovery University of Manchester and Manchester Metropolitan University; Consultant in Anaesthetics and Intensive Care Medicine, Manchester University NHS Foundation Trust<br>O'Connell, Sarah; The Christie National Health Service Foundation Trust, Department of Radiology<br>Taylor, Sally; The Christie School of Oncology, The Christie Patient Centred Research Team<br>Westwood, Thomas; The Christie National Health Service Foundation Trust, Department of Radiology<br>Whetton, Anthony; University of Manchester, Stoller Biomarker Discovery Centre; University of Manchester, Division of Cancer Sciences, Faculty of Biology, Medicine and Health<br>Yorke, Janelle; The Christie NHS Foundation Trust, Clinical Oncology; The University of Manchester School of Health Sciences, Christie Patient-

Centred Research, Division of Nursing, Midwifery & Social Work
Higham, Claire E; Christie Hospital NHS Foundation Trust, Department of Endocrinology; The University of Manchester, Manchester Academic Health Science Centre

Keywords: RADIOTHERAPY, Musculoskeletal disorders < ORTHOPAEDIC & TRAUMA SURGERY, Gynaecological oncology < GYNAECOLOGY, Clinical trials < THERAPEUTICS

SCHOLARONE™
Manuscripts

Title: RadBone: Bone Toxicity following Pelvic Radiotherapy. A prospective randomised controlled feasibility study evaluating a musculoskeletal health package in women with gynaecological cancers undergoing pelvic radiotherapy.

Authors:

Victoria Chatzimavridou-Grigoriadou[1], Lisa H Barraclough[2], Ivona Baricevic-Jones[3], Robert G Bristow[4], Martin Eden[5], Kate Haslett[2], Karen Johnson[2], Rohit Kochhar[6], Zoe Merchant[7], John Moore[8], Sarah O'Connell[6], Sally Taylor[9], Thomas Westwood[6], Anthony D Whetton[3], Janelle Yorke[9], Claire E Higham[1]

Affiliations:

[1]Department of Endocrinology, Christie Hospital NHS Foundation Trust, Manchester, UK; University of Manchester, Manchester Academic Health Science Centre, Manchester, UK

[2]Department of Clinical Oncology, Christie Hospital NHS Foundation Trust, Manchester, UK.

[3] Stoller Biomarker Discovery Centre, Division of Cancer Sciences, School of Medical Sciences, Faculty of Biology, Medicine and Health, University of Manchester, Manchester Academic Health Science Centre, Manchester, UK.

[4]Division of Cancer Sciences, The University of Manchester, Manchester, UK; CRUK Manchester Institute and Manchester Cancer Research Centre, Manchester, UK.

[5]Manchester Centre for Health Economics, The University of Manchester, Oxford Road, M13 9PL

[6]Department of Radiology, The Christie National Health Service Foundation Trust, Manchester, United Kingdom.

[7]Programme Lead for the Greater Manchester 'Prehab4Cancer and Recovery programme'/Highly Specialist Occupational Therapist. GM Cancer alliance hosted by the Christie NHS Foundation Trust.

[8]Consultant in Anaesthetics and Intensive Care Medicine, Manchester University NHS Foundation Trust. GM Cancer Clinical Director for Prehabilitation and Recovery. University of Manchester and Manchester Metropolitan University.

[9]The Christie Patient Centred Research Team, The Christie School of Oncology, The Christie NHS Foundation Trust, Wilmslow Road, Manchester, UK.

For peer review only

For peer review only - http://bmjopen.bmj.com/site/about/guidelines.xhtml

Corresponding authors:

- Dr Claire E Higham, DPhil, FRCP, Consultant Endocrinologist, Department of Endocrinology, Christie Hospital NHS Foundation Trust, Manchester, UK; University of Manchester, Manchester Academic Health Science Centre, Manchester, UK. claire.higham2@nhs.net
- Dr Victoria Chatzimavridou-Grigoriadou, MBBS, MRCP (UK), NIHR Academic Clinical Fellow in Endocrinology and Diabetes, Department of Endocrinology, Christie Hospital NHS Foundation Trust, Manchester, UK; University of Manchester, Manchester Academic Health Science Centre, Manchester, UK. victoriachgr@gmail.com

Abstract:

Introduction: Patients receiving radiotherapy are at risk of developing Radiotherapy Related Insufficiency Fractures (RRIFs), which are associated with increased morbidity and pose a significant burden to patients' quality of life and to the health system. Therefore, effective preventive techniques are urgently required. The RadBone randomized controlled trial (RCT) aims to determine the feasibility and acceptability of a musculoskeletal health package (MHP) intervention in women undergoing pelvic radiotherapy for gynaecological malignancies and to preliminary explore clinical effectiveness of the intervention.

Methods and Analysis: The RadBone RCT will evaluate the addition to standard care of a MHP consisting of a physical assessment of the musculoskeletal health, a three-month prehabilitation personalised exercise package, as well as an evaluation of the fracture risk and if required the prescription of appropriate bone treatment including calcium, vitamin D and - for high-risk individuals- bisphosphonates. Forty participants will be randomized in each group (MHP or observation) and will be followed for 18 months. The primary outcome of this RCT will be feasibility, including the eligibility, screening and recruitment rate, intervention fidelity and attrition rates; acceptability; and health economics. Clinical effectiveness and bone turnover markers will be evaluated as secondary outcomes.

Ethics and dissemination: This study has been approved by the Greater Manchester East Research Ethics Committee (Reference: 20/NW/0410, November 2020). The results will be published in peer reviewed journals, will be presented in national and international conferences, and will be communicated to relevant stakeholders. Moreover, a plain English report will be shared with the study participants, patients' organizations, and media.

Clinical trial registration: NCT04555317.

For peer review only - http://bmjopen.bmj.com/site/about/guidelines.xhtml

Keywords: radiotherapy, insufficiency fractures, musculoskeletal health, gynaecological cancer, randomised control trial.

Strengths and Limitations of this study

- The RadBone is the first randomized controlled trial to explore the feasibility and clinical effectiveness of a musculoskeletal health package aimed to prevent radiotherapy related insufficiency fractures (RRIFs).
- A feasibility economic evaluation will allow future assessment of this complex intervention's cost-effectiveness.
- Planned longitudinal proteomic analyses may reveal mechanistic insights and promising treatment targets.
- A prospectively published detailed protocol increases the transparency and allows for peer review of the methodology used.
- This study is not blinded and lacks an active comparator. Therefore, it is susceptible to performance and detection bias.

Word count: 2,982

For peer review only

Introduction

In 2015 there were 2.5 million people in the UK with a diagnosis of cancer and this number is expected to rise to 4 million by 2030[1]. As a result of the continuing improvement in early detection of disease and improved treatment efficacy, a significant proportion are living long beyond their cancer diagnosis. However, estimates suggest that currently over 500,000 people living with and beyond cancer have one or more physical or psychosocial consequences of their cancer or its treatment that affect their lives on a long-term basis. These consequences also have a substantial implication in terms of NHS resources.

Patients receiving radiotherapy are at risk of developing radiotherapy related bone toxicity, in particular radiotherapy related insufficiency fractures (RRIFs). Incidence of RRIFs following pelvic radiotherapy has been reported as between 1.7 and 89% and occurring between 3 to 20 months post radiotherapy. The wide variation in reported incidence depends on imaging modality and radiological reporting standards, symptomatic versus asymptomatic fractures, radiotherapy dose and underlying tumour type (reviewed in[2]). A recent meta-analysis of over 400 patients with RRIFs following pelvic radiotherapy for gynaecological cancers suggested an overall incidence of 14%[3]. Over 30 studies have been published since the 1990's describing more than 1000 patients with pelvic RRIFs. This literature is notable for being almost exclusively retrospective in nature, a sparsity of baseline assessment of bone density and fracture risk, the absence of Patient Reported Outcome Measures (PROMs) used to assess Quality of Life (QOL) and no primary preventative or secondary management intervention studies[4,5].

The devastating effects of osteoporotic fragility fractures on morbidity and mortality and the economic cost are well described[6]. Pelvic insufficiency fractures may also increase mortality [7] but these data reflect an elderly population with multiple co-morbidities and the applicability to the pelvic radiotherapy population is not well defined.

Although there are no pelvic RRIF studies reporting QOL as an outcome measure; clinical experience, patient radiotherapy support group reports and anecdotal commentary in the literature describe the anxiety, pain, reduced mobility and increased morbidity associated with these, with a number of patients requiring hospital admission for assessment and pain control[8]. Considering pelvic radiotherapy toxicities, including but not limited to RRIFs, more formal studies of QOL and PROMs are much needed[9].

Whilst a small number of studies, confirmed in a recent meta-analysis[3], suggest osteoporosis as a risk factor in pelvic RRIFs, unlike the strong evidence base for bisphosphonate use in primary and secondary prevention of fragility fractures, there is no such evidence for RRIFs[5]. A small non-controlled study demonstrated intravenous zolendronic acid administration prior to spinal radiotherapy led to a lower prevalence of radiotherapy bone toxicity than expected[10] and a single randomised prospective study in patients undergoing spinal radiotherapy for

For peer review only - http://bmjopen.bmj.com/site/about/guidelines.xhtml

metastatic disease demonstrated that intravenous zolendronic acid reduced urinary markers of collagen cross linking[11].

Contradictory data from animal studies around the protective effects of bisphosphonates on RRIFs limits our understanding of the pathophysiology and therapeutics of RRIFs. Animal studies using whole mouse radiation have demonstrated an early activation of bone resorption in the 5 days following low dose (2 Gy) of radiotherapy which was reduced by subcutaneous administration of risedronate immediately following irradiation[12]. In contrast, a focal radiation technique in mice (using a small animal radiation research platform or SARRP), arguably a more physiological representative method of irradiation, demonstrated that alendronic acid did not prevent the radiation induced trabecular bone loss but that this was prevented by blocking osteoblast apoptosis with PTH 1-34[13].

The RadBone is the first open-label prospective randomised controlled trial (RCT) to determine the feasibility and acceptability of a musculoskeletal health package (MHP) intervention in women undergoing pelvic radiotherapy for gynaecological malignancies and inform power calculations for a definitive RCT. Moreover, this feasibility trial will also explore potential implications on the incidence of RRIFs, quality of life and other clinical effectiveness and safety outcomes, as well as providing indicative estimates of the intervention's cost-effectiveness.

For peer review only

For peer review only - http://bmjopen.bmj.com/site/about/guidelines.xhtml

Methods and analysis:

Study Design

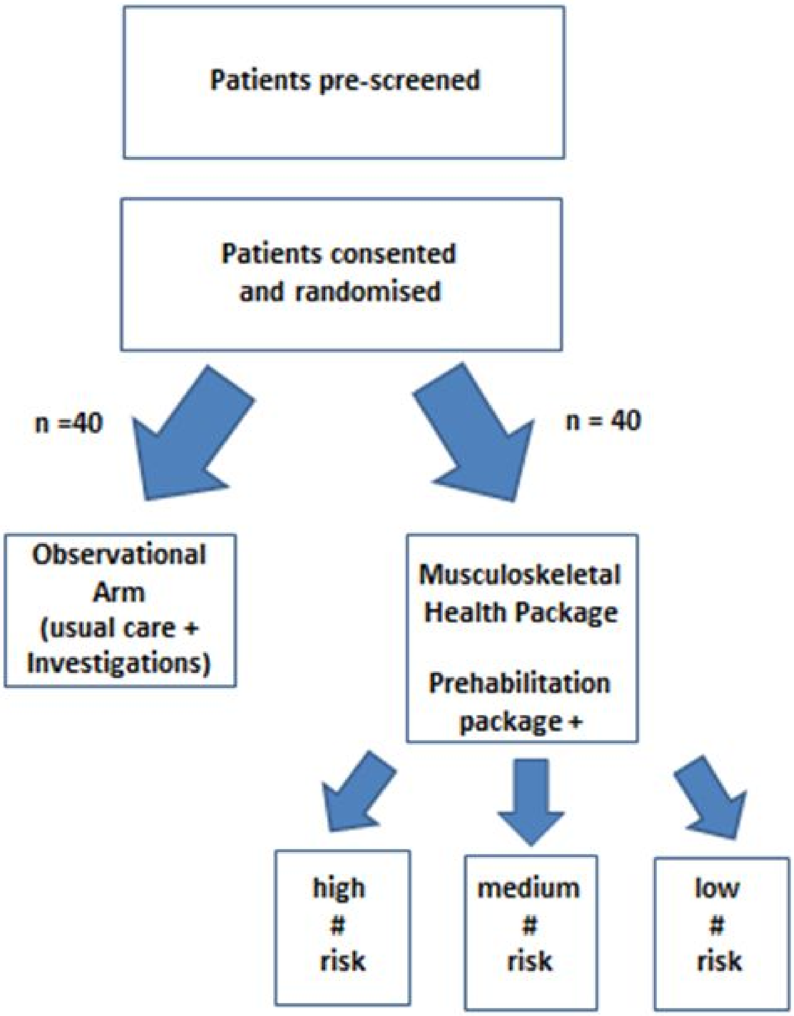

Study setting

The planned study is a prospective randomised controlled feasibility trial of eighty patients with gynaecological malignancy (cervical and endometrial) undergoing pelvic radiotherapy at the Christie Hospital NHS Foundation Trust in Manchester, UK (a tertiary referral Oncology centre).

For peer review only - http://bmjopen.bmj.com/site/about/guidelines.xhtml

Eligibility Criteria

Individuals aged over 18 years, with a histologically confirmed endometrial or cervical cancer undergoing potentially curative or adjuvant radiotherapy will be eligible, provided they are able and willing to provide an informed consent to participate.

The exclusion criteria are (i) age less than 18 years or greater than 85 years; (ii) pre-existing bone conditions such as osteoporosis treated with bisphosphonates in the previous 5 years, fibrous dysplasia, osteogenesis imperfecta, or other metabolic bone conditions; (iii) home address outside Greater Manchester; (iv) contraindication or intolerance of Magnetic Resonance scanning.

Interventions

Women undergoing radiotherapy for a gynaecological malignancy will be randomised to an observation (Ob) group and will receive standard assessment and care, following the current local clinical pathway, or an intervention group that will receive a “musculoskeletal health package” (MHP), in addition to standard assessment and care and will be followed for 18 months.

Patients randomised to the MHP arm will receive (i) a physical assessment of musculoskeletal health and a 3-month prehabilitation personalised exercise package as part of the Greater Manchester prehab4cancer program[14], (ii) a fracture risk assessment (FRAX) based on baseline dual-energy x-ray absorptiometry (DXA) bone mineral density (BMD), and (iii) treatment for bone health according to national UK recommendations i.e., standard of care for prevention of fragility fractures, by being subdivided into 3 groups (low risk, medium risk and high risk).

Patients with a normal BMD and a FRAX score below the National Osteoporosis Guideline Group (NOGG) recommended treatment line will be considered low risk. Medium risk is defined as osteopenia on the DXA, with FRAX score below the NOGG treatment line. Finally, those with osteopenia and a previous vertebral or hip fracture, or a FRAX score above the NOGG recommended treatment line will be considered high risk.

Low risk patients will be provided with a copy of the Royal Osteoporosis Society (ROS) “Healthy living for strong bones” leaflet. In addition to the leaflet, medium risk patients will receive calcium (1000 mg once daily) and vitamin D (800 IU per day) supplementation. The same interventions will be offered to high-risk patients, who will also undergo secondary osteoporosis screening (blood tests) and will receive oral alendronate 70mg once weekly, in

For peer review only - http://bmjopen.bmj.com/site/about/guidelines.xhtml

the absence of contraindications. Annual intravenous zolendronic acid infusion will be considered as an alternative where appropriate.

Those randomised to the observation arm will remain blinded to the results of the evaluations until the end of the study unless a fragility fracture or RRIF develops during the study.

Prehabilitation Exercise Programme (Prehab4cancer)

All patients randomised to the MHP arm of the study will be offered a bespoke prehabilitation exercise programme via the Prehab4cancer programme in Greater Manchester (http://www.prehab4cancer.co.uk/). The MHP arm patients will be referred to the Prehab4cancer team via electronic referral immediately following randomisation. Allocated patients will be individually assessed by the Prehab4cancer team according to their usual protocols and assigned an appropriate prehabilitation program. Duration of the programme is 12 weeks from the first assessment and participation will be encouraged, as tolerated.

Baseline and Follow-up Evaluation

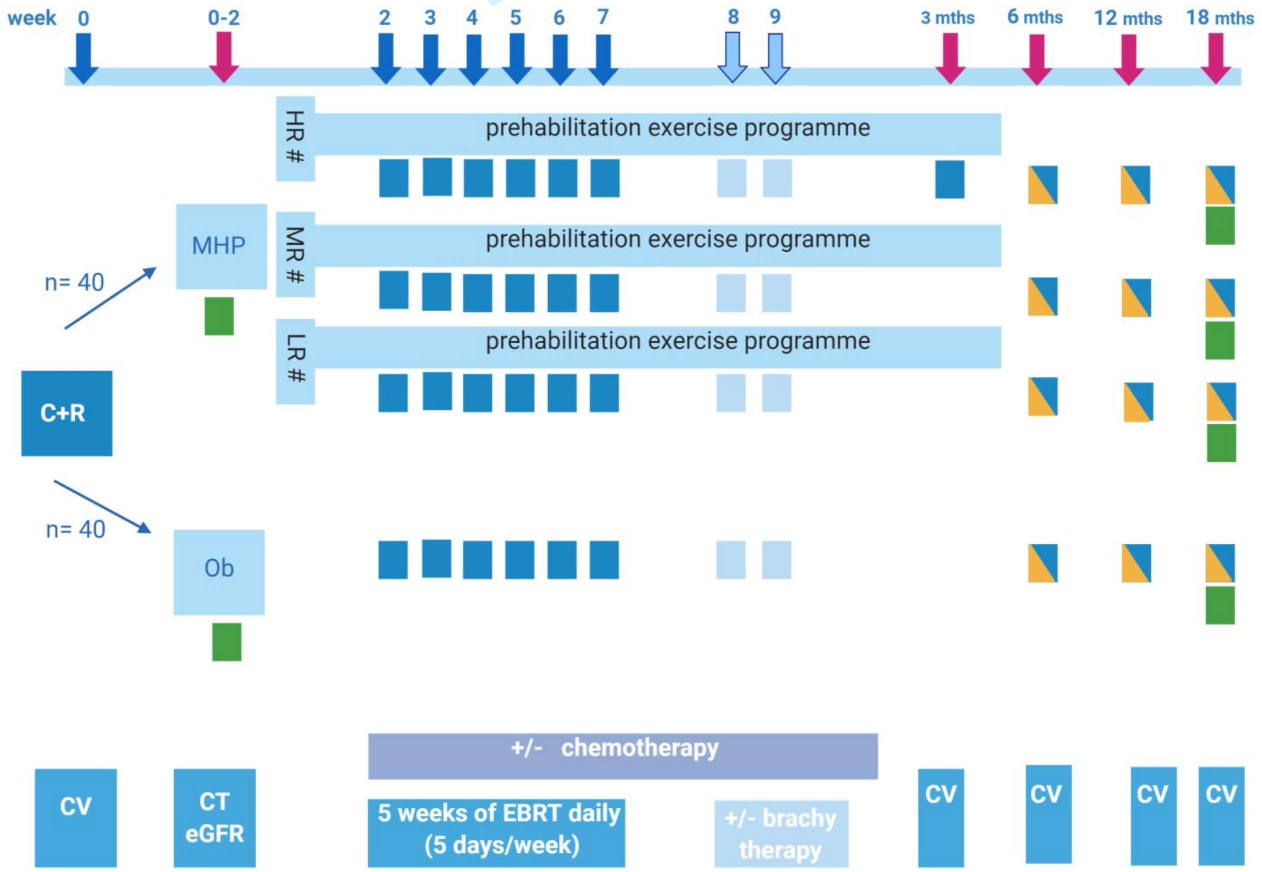

For peer review only - http://bmjopen.bmj.com/site/about/guidelines.xhtml

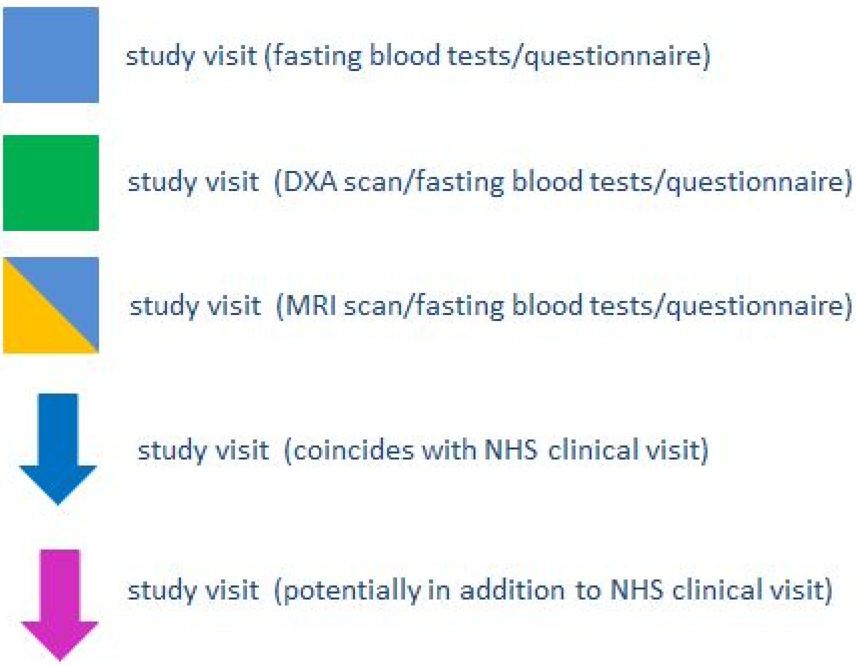

C+R = Consent and Randomisation, MHP= Musculoskeletal Health Package arm, Ob = observational arm HR = high risk, MR = medium risk, LR = low risk, CV = clinic visit, EBRT= External Beam Radiotherapy. NHS: National Health System

Baseline evaluations will include a bone health assessment with DXA BMD measurement and completion of a bone health questionnaire. PROMs will also be captured. Finally, fasting serum and plasma blood samples will also be collected.

At 6, 12 and 18 months post radiotherapy all patients will undergo a pelvic MRI assessment for RRIFs, PROMs assessment and fasting blood sampling. During the final visit, at 18 months, all patients will have a DXA BMD scan and physical assessment of their musculoskeletal health. If signs or symptoms compatible with a RRIF are described outside the study visits study participants will be assessed and managed following the current clinical pathways.

Imaging studies

DXA scans of the total hip, femoral neck, L1-L4 spine and TBS assessments will be performed on a single DXA scanner (Hologic Horizon A SN 300792M version 5.6.07 with TBS v.3.0.2 calibrated to the above scanner) at the Christie NHS Foundation Trust as per local protocol. These will be undertaken by two technicians trained in conducting DXA. Images will be reviewed, validated and interpreted by the lead investigator (CEH). The femoral neck BMD (g/cm^2) will be used in conjunction with a standardised DXA questionnaire to complete FRAX calculation.

For peer review only - http://bmjopen.bmj.com/site/about/guidelines.xhtml

Pelvic Magnetic Resonance Imaging (MRI) scans will be performed at 6, 12 and 18 months on a 1.5T MRI scanner at the Christie Hospital by trained radiographers in accordance with the study imaging protocol. Four pelvic sequences will be performed per patient (5mm slice thickness, field of view 400mm; coronal T1, coronal STIR (inversion time 150ms), axial T1 and axial STIR (inversion time 165 ms). These will correspond to routine follow-up scans where possible. All bone sequence scans will be dual reported by 2 consultant radiologists who will document the presence of fracture and their confidence in its presence, fracture location, fracture line, bone marrow oedema and other abnormalities.

Biochemical studies

Fasting blood tests will be performed at baseline, weekly during radiotherapy (visits 2 to 10, one day prior to chemotherapy if receiving) and at 6, 12 and 18 months in all patients. Patients allocated to the MHP High risk arm and started on oral bisphosphonate therapy will have an additional bone turnover marker blood test at 3 months to assess bisphosphonate efficacy. All samples will be taken simultaneously with routinely collected clinical blood samples where appropriate.

As part of the MHP intervention arm, blood will be sampled, analysed, and assessed at baseline, 6, 12 and 18 months for the measurement of full blood count, urea and electrolytes, liver function tests, parathyroid hormone, vitamin D, thyroid function test, oestradiol, HbA1c, procollagen type 1 amino-terminal propeptide (P1NP), and the beta-C-terminal telopeptide (CTx). Moreover, in the observation arm, serum samples will be collected, processed and stored at -80°C for batch analysis at the end of the study.

Additional fasting blood samples will be collected at all timepoints mentioned for longitudinal analysis of bone turnover markers and for proteomic analysis. These samples will be processed and stored at -80°C, following local standard operating procedures (SOPs), for batch analysis at the end of the study. Bone turnover markers will be evaluated using ELISA techniques and will include CTX, NTX, P1NP, osteocalcin, TRAcP5b and bone ALP.

Sequential Window Acquisition of all Theoretical Mass Spectra (SWATH-MS)

Proteomic analysis will be conducted at the Stoller Biomarker Discovery Centre, following local SOPs[15]. Samples will be analysed by a Data Independent Acquisition method known as SWATH-MS with a micro-flow LC-MS system comprising an Eksigent nanoLC 400 autosampler and an Eksigent nanoLC pump coupled to a SCIEX 6600 Triple-TOF mass spectrometer (68 min run-time). When SWATH maps are generated, the presence and abundance of plasma proteins will be quantified using published plasma reference libraries. Differential expression analysis will be used to identify candidate biomarkers using artificial intelligence approaches. Linear regression will be used to detect correlations with the presence of RRIFs and BMD.

For peer review only - http://bmjopen.bmj.com/site/about/guidelines.xhtml

Few longitudinal studies have tracked proteins of interest over the whole course of radiotherapy from pre-treatment baseline through to follow-up. We have undertaken one pilot that shows the potential value of this work[16]. Other studies that have investigated this have demonstrated distinguishing profiles with groups of approximately n=30. Two pre-radiotherapy baseline samples will be used to assess natural variation and comparison with the variance of measurements following radiotherapy and further comparison between the MHP and observation arm (n=40 per group).

Electronic data will be pseudoanonymised (coded) to protect the identity of the participants.

PROMS and Health Utilisation Proforma

PROMs will be collected either as electronic PROMS (using the myChristie, myHealth application) or paper-based PROMS at baseline 6, 12, and 18 months.

The evaluated PROMs will include the adapted pelvic patient-reported outcome version of the common terminology criteria for adverse events (PRO-CTCAE) assessment, the Short Musculoskeletal Function Assessment (SMFA) modified for lower limb, the 5-level version of the EuroQol tool (EQ-5D-5L) and a tailored Health Utilisation Proforma.

Criteria for discontinuing

Participants may decide to withdraw from the study at any time. Discontinuation of the study participants may occur as a result of investigator decision, safety concerns, and significant non-compliance to the protocol or incorrect enrolment. Reasons for discontinuation will be captured.

As this is a feasibility study, participants may decide to discontinue their participation in certain aspects of the study (for example declining the prehabilitation programme or deciding not to take recommended medications). The participants can continue with the study and the details will be captured in the case report form (CRF).

Outcomes

The primary outcomes for this feasibility study will inform the design and power calculations for a definitive UK multi-centre RCT. These are:

1) Eligibility and screening rate: proportion of patients eligible for the study from patient population
2) Recruitment and study group allocation rate: number and proportion of eligible patients recruited, randomised and allocated to appropriate study populations.

For peer review only - http://bmjopen.bmj.com/site/about/guidelines.xhtml

3) Intervention fidelity rate: number and proportion of patients completing the elements of the study (assessment visits, prehab exercise programme, prescribed medications, QOL questionnaire)
4) Attrition rate: number of patients lost to follow-up.
5) Patient and physician acceptability assessed with electronic questionnaires.
6) Health Economic Analysis: within-trial cost-effectiveness analysis to demonstrate feasibility of health economic data collection and analysis in a multi-centre RCT.

The secondary outcomes are:

1) Incidence of pelvic insufficiency fracture
2) Longitudinal change in BMD and fracture risk
3) Longitudinal change in biochemical markers of bone turnover
4) Longitudinal change in measured musculoskeletal health markers
5) Quality of Life
6) Incremental cost-effectiveness ratios: cost-per-QALY (quality-adjusted life-year) and cost-per-change in SMFA score

Exploratory Endpoints include identification of predictive markers of RRIFs (radiomic, proteomic, BMD) and exploratory measurement of proteomic biomarkers of bone turnover during pelvic radiotherapy.

Sample Size

No formal power calculation has been performed as this is a feasibility study. The study will collect initial data such as measures of location and variability for key outcome measures. It is recognised that in general, 30 patients are required in order to estimate such parameters[17]. For this study a total of 80 patients will be recruited and randomised with equal probability to either the MHP or observation arms (i.e. 40 per group). Assuming attrition rates of 15% per group, at least 30 should remain in each arm. This should be sufficient to assess the feasibility of a larger RCT study and estimate group means, standard deviations and percentages for key outcomes.

Recruitment

80 patients will be recruited over an 18-month period, approximately 4 patients per month. As this is a feasibility study there will be no interim analysis of study results.

Assignment of interventions

Consenting, eligible participants will be randomised to the MHP or observation group using a validated online service; sealedenvelope™ (https://www.sealedenvelope.com). A permuted

For peer review only - http://bmjopen.bmj.com/site/about/guidelines.xhtml

block (block size:4) randomisation protocol will be utilised with a 1:1 allocation (MHP to observation arm).

Data Collection, management and analysis

Statistical and Health Economic Analysis

As this is a feasibility study, it will not involve hypothesis testing to identify whether the intervention has had an impact. Instead, data analysis will be descriptive, focusing on the percentage of patients in each group developing RRIFs and risk factors for this. Means and a measure of variation will be calculated for each secondary outcome. These data, along with estimates of recruitment and attrition rates, will help inform a power calculation for the definitive trial.

A within-trial cost-effectiveness analysis[18] will be undertaken from the perspective of the UK National Health Service (NHS). Cost data for the intervention arm will reflect resource use associated with the musculoskeletal health package and treatment costs for both the control and intervention arm will be taken into account. Resource use will be extracted from patient records and the health care utilisation proforma. Relevant sources (e.g., NHS reference costs) will be used to identify unit costs. Health related quality of life (HRQL) scores will be generated using the EQ-5D-5L at baseline and at each of the three follow-up time points (6, 12, 18 months).

A descriptive analysis of the costs and outcomes data will be completed focusing on: a. whether the EQ-5D-5L and SMFA are able to adequately capture differences in health status before and after implementation of the musculoskeletal health package and across both treatment arms of the study; b. whether the resource-use survey is able to record data necessary to enable a full cost-effectiveness analysis; c. the nature of missing data for the EQ-5D-5L, SMFA and resource-use survey to assess responses, sensitivity, and any patterns within the missing data.

A within-trial cost-effectiveness analysis will be conducted to provide an indicative estimate of cost-effectiveness. Between-arm differences in costs and outcomes will be expressed as an incremental cost-effectiveness ratio (ICERs): the cost per QALY gained from the intervention compared to usual care. ICERs will also be calculated using the SMFA in an additional scenario analysis.

Trial oversight

An internal trial management group will be convened for the study, consisting of the chief investigator, project manager, Clinical Trials administrator, research nurse and a representative of the research and innovation division (R+I) as core members. The group will

For peer review only - http://bmjopen.bmj.com/site/about/guidelines.xhtml

meet monthly. The study sponsor (Christie Hospital NHS Foundation Trust) will monitor the conduct of the trial.

Patient and Public Involvement

This protocol was developed with the participation of the Christie pelvic radiotherapy user group and supported by the Pelvic Radiation Disease Association (PRDA).

Ethics and Dissemination

This study has been approved by GM East Research Ethics Committee in November 2020 (REC reference 20/NW/0410) and is registered at clinicaltrials.gov (ClinicalTrials.gov Identifier: NCT04555317). The study opened for recruitment in May 2021. The results of this study will be published in peer reviewed journals, will be presented in national and international conferences, and will be communicated to relevant stakeholders. Moreover, a plain English report will be shared with the study participants, patients' organizations, and media.

Authors' contributions: CEH developed the protocol, MRC application and ethics application for the study. RB is the MRC-CARP academic partner to CEH and contributed to the study design and protocol.

KJ, LHB, KH contributed to the development of the gynae-oncology aspects

RK, SO'C, TW contributed to the development of the MRI radiology aspects

ZM, JM contributed to the development of the Prehab4Cancer aspects

ST, JY contributed to the PROMS development

ME contributed to the Health Economic Analysis

ADW, IBJ contributed to the proteomic and data analysis

VCG and CEH prepared the manuscript

All authors: critically reviewed and commented on the manuscript.

Funding statement: This work is partially supported by an MRC-NIHR Clinical Academic Research Partnership) award: grant number MR/T024887/1. Funding is being sought for Health Economic Analysis, QOL and PROMS development and longitudinal proteomic analysis. Equipment used in the Stoller Biomarker Discovery Centre is funded by a donation received from the Stoller Charitable Trust and a research grant awarded by the Medical Research Council (MR/M008959/1). MRC/EPSRC Molecular Pathology Node provided additional

For peer review only

financial support by a grant from the Medical Research Council and Engineering & Physical Sciences Research Council (MR/N00583X/1).

Competing interests statement. No competing interests

Full references

1. Available from: https://www.macmillan.org.uk/_images/cancer-statistics-factsheet_tcm9-260514.pdf [Accessed June 2021]
2. Higham CE, Faithfull S. Bone Health and Pelvic Radiotherapy. Clin Oncol (R Coll Radiol). 2015;27(11):668-678. doi:10.1016/j.clon.2015.07.006
3. Sapienza LG, Salcedo MP, Ning MS, et al. Pelvic Insufficiency Fractures After External Beam Radiation Therapy for Gynecologic Cancers: A Meta-analysis and Meta-regression of 3929 Patients. Int J Radiat Oncol Biol Phys. 2020;106(3):475-484. doi:10.1016/j.ijrobp.2019.09.012
4. Gebauer J, Higham C, Langer T, Denzer C, Brabant G. Long-Term Endocrine and Metabolic Consequences of Cancer Treatment: A Systematic Review. Endocr Rev. 2019;40(3):711-767. doi:10.1210/er.2018-00092
5. van den Blink QU, Garcez K, Henson CC, Davidson SE, Higham CE. Pharmacological interventions for the prevention of insufficiency fractures and avascular necrosis associated with pelvic radiotherapy in adults. Cochrane Database Syst Rev. 2018;4(4):CD010604. Published 2018 Apr 23. doi:10.1002/14651858.CD010604.pub2.
6. Center JR, Nguyen TV, Schneider D, Sambrook PN, Eisman JA. Mortality after all major types of osteoporotic fracture in men and women: an observational study. Lancet. 1999;353(9156):878-882. doi:10.1016/S0140-6736(98)09075-8
7. Taillandier J, Langue F, Alemanni M, Taillandier-Heriche E. Mortality and functional outcomes of pelvic insufficiency fractures in older patients. Joint Bone Spine. 2003;70(4):287-289. doi:10.1016/s1297-319x(03)00015-0
8. Oh D, Huh SJ, Nam H, et al. Pelvic insufficiency fracture after pelvic radiotherapy for cervical cancer: analysis of risk factors. Int J Radiat Oncol Biol Phys. 2008;70(4):1183-1188. doi:10.1016/j.ijrobp.2007.08.005
9. Holch P, Pini S, Henry AM, et al. eRAPID electronic patient self-Reporting of Adverse-events: Patient Information and aDvice: a pilot study protocol in pelvic radiotherapy. Pilot Feasibility Stud. 2018;4:110. Published 2018 Jun 5. doi:10.1186/s40814-018-0304-6
10. Pichon B, Campion L, Delpon G, et al. High-Dose Hypofractionated Radiation Therapy for Noncompressive Vertebral Metastases in Combination With Zoledronate: A Phase 1 Study. Int J Radiat Oncol Biol Phys. 2016;96(4):840-847. doi:10.1016/j.ijrobp.2016.07.027
11. Gierloff M, Reutemann M, Gülses A, Niehoff P, Wiltfang J, Açil Y. Effects of zoledronate on the radiation-induced collagen breakdown: a prospective randomized clinical trial. Clin Transl Oncol. 2015;17(6):454-461. doi:10.1007/s12094-014-1257-8
12. Willey JS, Livingston EW, Robbins ME, et al. Risedronate prevents early radiation-induced osteoporosis in mice at multiple skeletal locations. Bone. 2010;46(1):101-111. doi:10.1016/j.bone.2009.09.002
13. Chandra A, Lin T, Tribble MB, et al. PTH1-34 alleviates radiotherapy-induced local bone loss by improving osteoblast and osteocyte survival. Bone. 2014;67:33-40. doi:10.1016/j.bone.2014.06.030
14. Moore J, Merchant Z, Rowlinson K, et al. Implementing a system-wide cancer prehabilitation programme: The journey of Greater Manchester's 'Prehab4cancer'. Eur J Surg Oncol. 2021;47(3 Pt A):524-532. doi:10.1016/j.ejso.2020.04.042
15. Geary B, Walker MJ, Snow JT, et al. Identification of a Biomarker Panel for Early Detection of Lung Cancer Patients. J Proteome Res. 2019;18(9):3369-3382. doi:10.1021/acs.jproteome.9b00287
16. Walker MJ, Zhou C, Backen A, et al. Discovery and Validation of Predictive Biomarkers of Survival for Non-small Cell Lung Cancer Patients Undergoing Radical Radiotherapy: Two Proteins With Predictive Value. EBioMedicine. 2015;2(8):841-850. Published 2015 Jun 19. doi:10.1016/j.ebiom.2015.06.013

For peer review only - http://bmjopen.bmj.com/site/about/guidelines.xhtml

17. Billingham SA, Whitehead AL, Julious SA. An audit of sample sizes for pilot and feasibility trials being undertaken in the United Kingdom registered in the United Kingdom Clinical Research Network database. BMC Med Res Methodol. 2013;13:104. Published 2013 Aug 20. doi:10.1186/1471-2288-13-104
18. Drummond MF, Sculpher MJ, Claxton K, Stoddart GL, Torrance GW. Methods for the Economic Evaluation of Health Care Programmes. 4th ed. Oxford: Oxford University Press, Oxford. <https://books.google.co.uk/books?id=lvWACgAAQBAJ>

For peer review only

For peer review only - http://bmjopen.bmj.com/site/about/guidelines.xhtml

Study Design

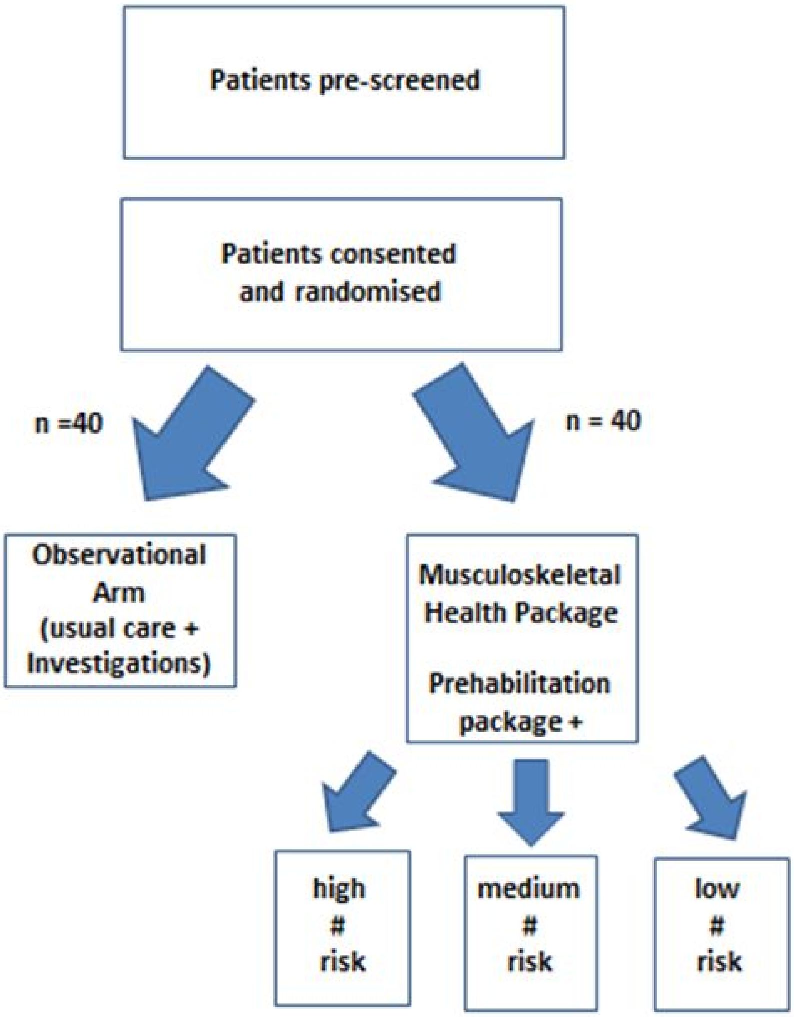

Baseline and Follow-up Evaluation

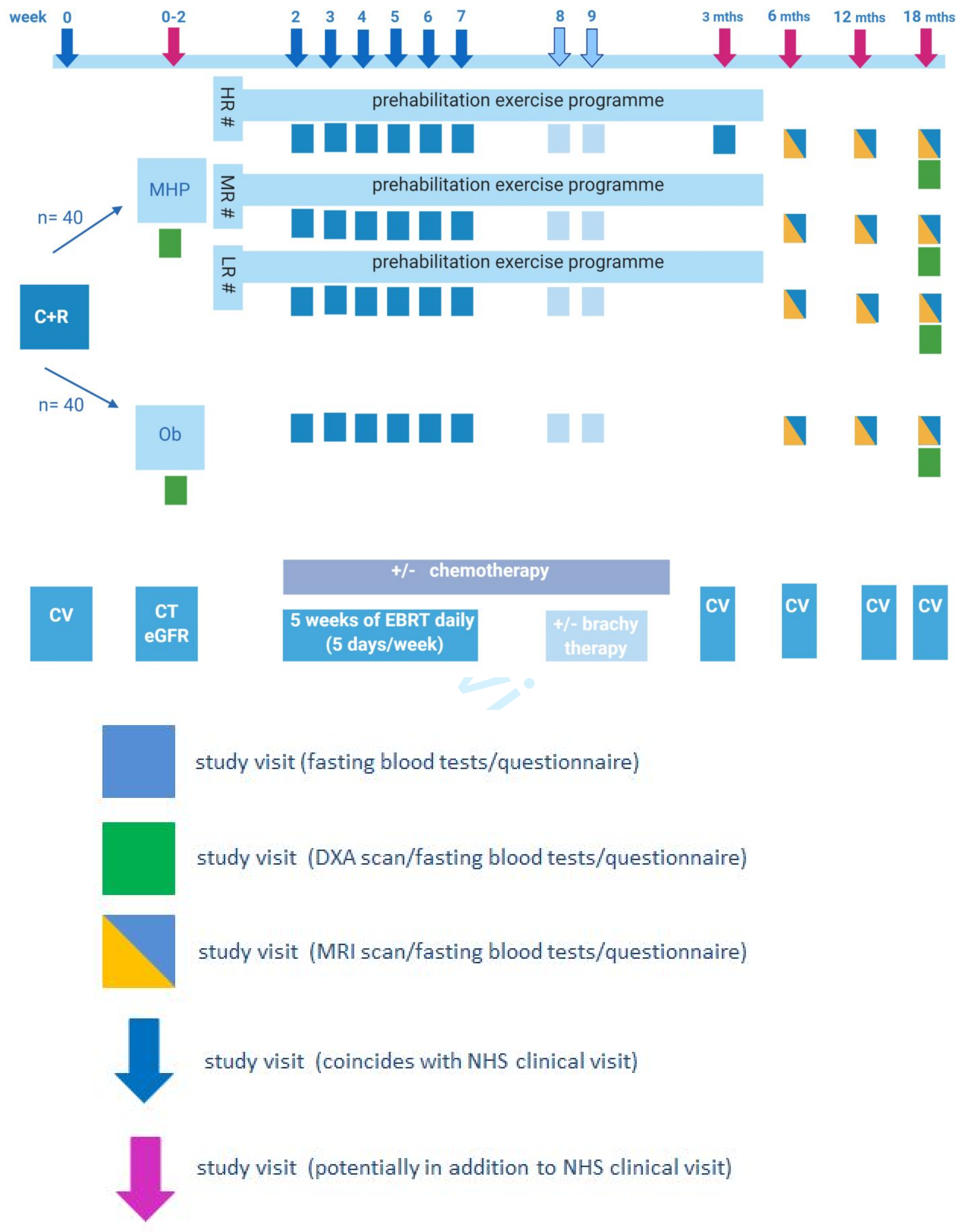

C+R = Consent and Randomisation, MHP= Musculoskeletal Health Package arm, Ob = observational arm HR = high risk, MR = medium risk, LR = low risk, CV = clinic visit, EBRT= External Beam Radiotherapy. NHS: National Health System

For peer review only

Reporting checklist for protocol of a clinical trial.

Based on the SPIRIT guidelines.

Instructions to authors

Complete this checklist by entering the page numbers from your manuscript where readers will find each of the items listed below.

Your article may not currently address all the items on the checklist. Please modify your text to include the missing information. If you are certain that an item does not apply, please write "n/a" and provide a short explanation.

Upload your completed checklist as an extra file when you submit to a journal.

In your methods section, say that you used the SPIRITreporting guidelines, and cite them as:

Chan A-W, Tetzlaff JM, Gøtzsche PC, Altman DG, Mann H, Berlin J, Dickersin K, Hróbjartsson A, Schulz KF, Parulekar WR, Krleža-Jerić K, Laupacis A, Moher D. SPIRIT 2013 Explanation and Elaboration: Guidance for protocols of clinical trials. BMJ. 2013;346:e7586

		Reporting Item	Page Number
**Administrative information**			
Title	#1	Descriptive title identifying the study design, population, interventions, and, if applicable, trial acronym	1
Trial registration	#2a	Trial identifier and registry name. If not yet registered,	2

For peer review only

		name of intended registry	
Trial registration: data set	#2b	All items from the World Health Organization Trial Registration Data Set	6-14
Protocol version	#3	Date and version identifier	N/A
Funding	#4	Sources and types of financial, material, and other support	14,15
Roles and responsibilities: contributorship	#5a	Names, affiliations, and roles of protocol contributors	14
Roles and responsibilities: sponsor contact information	#5b	Name and contact information for the trial sponsor	13,14
Roles and responsibilities: sponsor and funder	#5c	Role of study sponsor and funders, if any, in study design; collection, management, analysis, and interpretation of data; writing of the report; and the decision to submit the report for publication, including whether they will have ultimate authority over any of these activities	13,14
Roles and responsibilities: committees	#5d	Composition, roles, and responsibilities of the coordinating centre, steering committee, endpoint adjudication committee, data management team, and other individuals or groups overseeing the trial, if applicable (see Item 21a for data monitoring committee)	13,14

Introduction

For peer review only

Background and rationale	#6a	Description of research question and justification for undertaking the trial, including summary of relevant studies (published and unpublished) examining benefits and harms for each intervention	5
Background and rationale: choice of comparators	#6b	Explanation for choice of comparators	5
Objectives	#7	Specific objectives or hypotheses	4,5
Trial design	#8	Description of trial design including type of trial (eg, parallel group, crossover, factorial, single group), allocation ratio, and framework (eg, superiority, equivalence, non-inferiority, exploratory)	5
**Methods: Participants, interventions, and outcomes**			
Study setting	#9	Description of study settings (eg, community clinic, academic hospital) and list of countries where data will be collected. Reference to where list of study sites can be obtained	6
Eligibility criteria	#10	Inclusion and exclusion criteria for participants. If applicable, eligibility criteria for study centres and individuals who will perform the interventions (eg,	7

For peer review only

		surgeons, psychotherapists)	
Interventions: description	#11a	Interventions for each group with sufficient detail to allow replication, including how and when they will be administered	7,8
Interventions: modifications	#11b	Criteria for discontinuing or modifying allocated interventions for a given trial participant (eg, drug dose change in response to harms, participant request, or improving / worsening disease)	11
Interventions: adherance	#11c	Strategies to improve adherence to intervention protocols, and any procedures for monitoring adherence (eg, drug tablet return; laboratory tests)	N/A
Interventions: concomitant care	#11d	Relevant concomitant care and interventions that are permitted or prohibited during the trial	7,8
Outcomes	#12	Primary, secondary, and other outcomes, including the specific measurement variable (eg, systolic blood pressure), analysis metric (eg, change from baseline, final value, time to event), method of aggregation (eg, median, proportion), and time point for each outcome. Explanation of the clinical relevance of chosen efficacy and harm outcomes is strongly recommended	11,12
Participant timeline	#13	Time schedule of enrolment, interventions (including any run-ins and washouts), assessments, and visits for participants. A schematic diagram is highly recommended (see Figure)	8-11

For peer review only

Sample size	#14	Estimated number of participants needed to achieve study objectives and how it was determined, including clinical and statistical assumptions supporting any sample size calculations	12
Recruitment	#15	Strategies for achieving adequate participant enrolment to reach target sample size	12
**Methods: Assignment of interventions (for controlled trials)**			
Allocation: sequence generation	#16a	Method of generating the allocation sequence (eg, computer-generated random numbers), and list of any factors for stratification. To reduce predictability of a random sequence, details of any planned restriction (eg, blocking) should be provided in a separate document that is unavailable to those who enrol participants or assign interventions	12,13
Allocation concealment mechanism	#16b	Mechanism of implementing the allocation sequence (eg, central telephone; sequentially numbered, opaque, sealed envelopes), describing any steps to conceal the sequence until interventions are assigned	12,13
Allocation: implementation	#16c	Who will generate the allocation sequence, who will enrol participants, and who will assign participants to interventions	12,13

For peer review only

Blinding (masking)	#17a	Who will be blinded after assignment to interventions (eg, trial participants, care providers, outcome assessors, data analysts), and how	5
Blinding (masking): emergency unblinding	#17b	If blinded, circumstances under which unblinding is permissible, and procedure for revealing a participant's allocated intervention during the trial	N/a
**Methods: Data collection, management, and analysis**			
Data collection plan	#18a	Plans for assessment and collection of outcome, baseline, and other trial data, including any related processes to promote data quality (eg, duplicate measurements, training of assessors) and a description of study instruments (eg, questionnaires, laboratory tests) along with their reliability and validity, if known. Reference to where data collection forms can be found, if not in the protocol	9-11
Data collection plan: retention	#18b	Plans to promote participant retention and complete follow-up, including list of any outcome data to be collected for participants who discontinue or deviate from intervention protocols	11
Data management	#19	Plans for data entry, coding, security, and storage, including any related processes to promote data quality (eg, double data entry; range checks for data values).	13

For peer review only

		Reference to where details of data management procedures can be found, if not in the protocol	
Statistics: outcomes	#20a	Statistical methods for analysing primary and secondary outcomes. Reference to where other details of the statistical analysis plan can be found, if not in the protocol	13
Statistics: additional analyses	#20b	Methods for any additional analyses (eg, subgroup and adjusted analyses)	13
Statistics: analysis population and missing data	#20c	Definition of analysis population relating to protocol non-adherence (eg, as randomised analysis), and any statistical methods to handle missing data (eg, multiple imputation)	13
**Methods: Monitoring**			
Data monitoring: formal committee	#21a	Composition of data monitoring committee (DMC); summary of its role and reporting structure; statement of whether it is independent from the sponsor and competing interests; and reference to where further details about its charter can be found, if not in the protocol. Alternatively, an explanation of why a DMC is not needed	13,14
Data monitoring: interim analysis	#21b	Description of any interim analyses and stopping guidelines, including who will have access to these interim results and make the final decision to terminate the trial	12
Harms	#22	Plans for collecting, assessing, reporting, and managing solicited and spontaneously reported adverse events and other unintended effects of trial interventions or trial	11

For peer review only

		conduct	
Auditing	#23	Frequency and procedures for auditing trial conduct, if any, and whether the process will be independent from investigators and the sponsor	14
**Ethics and dissemination**			
Research ethics approval	#24	Plans for seeking research ethics committee / institutional review board (REC / IRB) approval	2
Protocol amendments	#25	Plans for communicating important protocol modifications (eg, changes to eligibility criteria, outcomes, analyses) to relevant parties (eg, investigators, REC / IRBs, trial participants, trial registries, journals, regulators)	13,14
Consent or assent	#26a	Who will obtain informed consent or assent from potential trial participants or authorised surrogates, and how (see Item 32)	7
Consent or assent: ancillary studies	#26b	Additional consent provisions for collection and use of participant data and biological specimens in ancillary studies, if applicable	N/A
Confidentiality	#27	How personal information about potential and enrolled participants will be collected, shared, and maintained in order to protect confidentiality before, during, and after the trial	13
Declaration of	#28	Financial and other competing interests for principal	14,15

For peer review only

interests		investigators for the overall trial and each study site	
Data access	#29	Statement of who will have access to the final trial dataset, and disclosure of contractual agreements that limit such access for investigators	TBC
Ancillary and post trial care	#30	Provisions, if any, for ancillary and post-trial care, and for compensation to those who suffer harm from trial participation	N/A
Dissemination policy: trial results	#31a	Plans for investigators and sponsor to communicate trial results to participants, healthcare professionals, the public, and other relevant groups (eg, via publication, reporting in results databases, or other data sharing arrangements), including any publication restrictions	14
Dissemination policy: authorship	#31b	Authorship eligibility guidelines and any intended use of professional writers	14
Dissemination policy: reproducible research	#31c	Plans, if any, for granting public access to the full protocol, participant-level dataset, and statistical code	14
**Appendices**			
Informed consent materials	#32	Model consent form and other related documentation given to participants and authorised surrogates	TBC
Biological specimens	#33	Plans for collection, laboratory evaluation, and storage of biological specimens for genetic or molecular analysis in the current trial and for future use in ancillary studies, if applicable	N/A

For peer review only

None The SPIRIT Explanation and Elaboration paper is distributed under the terms of the Creative Commons Attribution License CC-BY-NC. This checklist can be completed online using https://www.goodreports.org/, a tool made by the EQUATOR Network in collaboration with Penelope.ai

For peer review only

BMJ Open

RadBone: Bone Toxicity following Pelvic Radiotherapy. A prospective randomised controlled feasibility study evaluating a musculoskeletal health package in women with gynaecological cancers undergoing pelvic radiotherapy.

Journal:	*BMJ Open*
Manuscript ID	bmjopen-2021-056600.R1
Article Type:	Protocol
Date Submitted by the Author:	09-Mar-2022

Complete List of Authors:	Chatzimavridou Grigoriadou, Victoria; Christie Hospital NHS Foundation Trust, Department of Endocrinology; University of Manchester, Manchester Academic Health Science Centre<br>Barraclough, Lisa H; Christie Hospital NHS Foundation Trust, Department of Clinical Oncology<br>Baricevic-Jones, Ivona; Manchester Academic Health Science Centre, Stoller Biomarker Discovery Centre, Division of Cancer Sciences, School of Medical Sciences, Faculty of Biology, Medicine and Health, University of Manchester<br>Bristow, Robert; University of Manchester, Manchester Cancer Research Centre<br>Eden, Martin; The University of Manchester Manchester Centre for Health Economics<br>Haslett, Kate; Christie Hospital NHS Foundation Trust, Department of Clinical Oncology<br>Johnson, Karen; Christie Hospital NHS Foundation Trust, Department of Clinical Oncology<br>Kochhar, Rohit; The Christie National Health Service Foundation Trust, Department of Radiology<br>Merchant, Zoe; Programme Lead for the Greater Manchester 'Prehab4Cancer and Recovery programme'/Highly Specialist Occupational Therapist. GM Cancer alliance hosted by the Christie NHS Foundation Trust.<br>Moore, John; GM Cancer Clinical Director for Prehabilitation and Recovery University of Manchester and Manchester Metropolitan University; Consultant in Anaesthetics and Intensive Care Medicine, Manchester University NHS Foundation Trust<br>O'Connell, Sarah; The Christie National Health Service Foundation Trust, Department of Radiology<br>Taylor, Sally; The Christie School of Oncology, The Christie Patient Centred Research Team<br>Westwood, Thomas; The Christie National Health Service Foundation Trust, Department of Radiology<br>Whetton, Anthony; University of Manchester, Stoller Biomarker Discovery Centre; University of Manchester, Division of Cancer Sciences, Faculty of Biology, Medicine and Health<br>Yorke, Janelle; The Christie NHS Foundation Trust, Clinical Oncology; The University of Manchester School of Health Sciences, Christie Patient-

	Centred Research, Division of Nursing, Midwifery & Social Work<br>Higham, Claire E; Christie Hospital NHS Foundation Trust, Department of Endocrinology; The University of Manchester, Manchester Academic Health Science Centre
**Primary Subject Heading**:	Diabetes and endocrinology
Secondary Subject Heading:	Oncology
Keywords:	RADIOTHERAPY, Musculoskeletal disorders < ORTHOPAEDIC & TRAUMA SURGERY, Gynaecological oncology < GYNAECOLOGY, Clinical trials < THERAPEUTICS

Title: RadBone: Bone Toxicity following Pelvic Radiotherapy. A prospective randomised controlled feasibility study evaluating a musculoskeletal health package in women with gynaecological cancers undergoing pelvic radiotherapy.

Authors:

Victoria Chatzimavridou-Grigoriadou[1], Lisa H Barraclough[2], Ivona Baricevic-Jones[3], Robert G Bristow[4], Martin Eden[5], Kate Haslett[2], Karen Johnson[2], Rohit Kochhar[6], Zoe Merchant[7], John Moore[8], Sarah O'Connell[6], Sally Taylor[9], Thomas Westwood[6], Anthony D Whetton[3], Janelle Yorke[9], Claire E Higham[1]

Affiliations:

[1]Department of Endocrinology, Christie Hospital NHS Foundation Trust, Manchester, UK; University of Manchester, Manchester Academic Health Science Centre, Manchester, UK

[2]Department of Clinical Oncology, Christie Hospital NHS Foundation Trust, Manchester, UK.

[3] Stoller Biomarker Discovery Centre, Division of Cancer Sciences, School of Medical Sciences, Faculty of Biology, Medicine and Health, University of Manchester, Manchester Academic Health Science Centre, Manchester, UK.

[4]Division of Cancer Sciences, The University of Manchester, Manchester, UK; CRUK Manchester Institute and Manchester Cancer Research Centre, Manchester, UK.

[5]Manchester Centre for Health Economics, The University of Manchester, Oxford Road, M13 9PL

[6]Department of Radiology, The Christie National Health Service Foundation Trust, Manchester, United Kingdom.

[7]Programme Lead for the Greater Manchester 'Prehab4Cancer and Recovery programme'/Highly Specialist Occupational Therapist. GM Cancer alliance hosted by the Christie NHS Foundation Trust.

[8]Consultant in Anaesthetics and Intensive Care Medicine, Manchester University NHS Foundation Trust. GM Cancer Clinical Director for Prehabilitation and Recovery. University of Manchester and Manchester Metropolitan University.

[9]The Christie Patient Centred Research Team, The Christie School of Oncology, The Christie NHS Foundation Trust, Wilmslow Road, Manchester, UK.

For peer review only - http://bmjopen.bmj.com/site/about/guidelines.xhtml

Corresponding authors:

- Dr Claire E Higham, DPhil, FRCP, Consultant Endocrinologist, Department of Endocrinology, Christie Hospital NHS Foundation Trust, Manchester, UK; University of Manchester, Manchester Academic Health Science Centre, Manchester, UK. claire.higham2@nhs.net
- Dr Victoria Chatzimavridou-Grigoriadou, MBBS, MRCP (UK), NIHR Academic Clinical Fellow in Endocrinology and Diabetes, Department of Endocrinology, Christie Hospital NHS Foundation Trust, Manchester, UK; University of Manchester, Manchester Academic Health Science Centre, Manchester, UK. victoriachgr@gmail.com

Abstract:

Introduction: Patients receiving radiotherapy are at risk of developing Radiotherapy Related Insufficiency Fractures (RRIFs), which are associated with increased morbidity and pose a significant burden to patients' quality of life and to the health system. Therefore, effective preventive techniques are urgently required. The RadBone randomized controlled trial (RCT) aims to determine the feasibility and acceptability of a musculoskeletal health package (MHP) intervention in women undergoing pelvic radiotherapy for gynaecological malignancies and to preliminary explore clinical effectiveness of the intervention.

Methods and Analysis: The RadBone RCT will evaluate the addition to standard care of a MHP consisting of a physical assessment of the musculoskeletal health, a three-month prehabilitation personalised exercise package, as well as an evaluation of the fracture risk and if required the prescription of appropriate bone treatment including calcium, vitamin D and -for high-risk individuals- bisphosphonates. Forty participants will be randomized in each group (MHP or observation) and will be followed for 18 months. The primary outcome of this RCT will be feasibility, including the eligibility, screening and recruitment rate, intervention fidelity and attrition rates; acceptability; and health economics. Clinical effectiveness and bone turnover markers will be evaluated as secondary outcomes.

Ethics and dissemination: This study has been approved by the Greater Manchester East Research Ethics Committee (Reference: 20/NW/0410, November 2020). The results will be published in peer reviewed journals, will be presented in national and international conferences, and will be communicated to relevant stakeholders. Moreover, a plain English report will be shared with the study participants, patients' organizations, and media.

Clinical trial registration: NCT04555317.

For peer review only

For peer review only - http://bmjopen.bmj.com/site/about/guidelines.xhtml

Keywords: radiotherapy, insufficiency fractures, musculoskeletal health, gynaecological cancer, randomised control trial.

Strengths and Limitations of this study

- The RadBone is the first randomized controlled trial to assess a musculoskeletal health package aimed to prevent radiotherapy related insufficiency fractures (RRIFs).
- A feasibility economic evaluation will allow future assessment of this complex intervention's cost-effectiveness.
- Planned longitudinal proteomic analyses may reveal mechanistic insights and promising treatment targets.
- A prospectively published detailed protocol increases the transparency and allows for peer review of the methodology used.
- This study is not blinded and lacks an active comparator, hence, it is susceptible to performance and detection bias.

Word count: 3,740

For peer review only - http://bmjopen.bmj.com/site/about/guidelines.xhtml

Introduction

In 2015 there were 2.5 million people in the UK with a diagnosis of cancer and this number is expected to rise to 4 million by 2030[1]. As a result of the continuing improvement in early detection of disease and improved treatment efficacy, a significant proportion are living long beyond their cancer diagnosis. However, estimates suggest that currently over 500,000 people living with and beyond cancer have one or more physical or psychosocial consequences of their cancer or its treatment that affect their lives on a long-term basis. These consequences also have a substantial implication in terms of NHS resources.

Patients receiving radiotherapy are at risk of developing radiotherapy related bone toxicity, in particular radiotherapy related insufficiency fractures (RRIFs). Incidence of RRIFs following pelvic radiotherapy has been reported as between 1.7 and 89% and occurring between 3 to 20 months post radiotherapy. The wide variation in reported incidence depends on imaging modality and radiological reporting standards, symptomatic versus asymptomatic fractures, radiotherapy dose and underlying tumour type (reviewed in[2]). A recent meta-analysis of over 400 patients with RRIFs following pelvic radiotherapy for gynaecological cancers suggested an overall incidence of 14%[3]. Over 30 studies have been published since the 1990's describing more than 1000 patients with pelvic RRIFs. This literature is notable for being almost exclusively retrospective in nature, a sparsity of baseline assessment of bone density and fracture risk, the absence of Patient Reported Outcome Measures (PROMs) used to assess Quality of Life (QOL) and no primary preventative or secondary management intervention studies[4,5].

For peer review only

The devastating effects of osteoporotic fragility fractures on morbidity and mortality and the economic cost are well described[6]. Pelvic insufficiency fractures may also increase mortality [7] but these data reflect an elderly population with multiple co-morbidities and the applicability to the pelvic radiotherapy population is not well defined. In addition, there are no pelvic RRIF studies reporting QOL as an outcome measure. However, the anxiety, pain, reduced mobility and increased morbidity associated with these has been described, with a number of patients requiring hospital admission for assessment and pain control[8]. Therefore, formal studies of QOL and PROMs are much needed, considering the wide range of pelvic radiotherapy toxicities[9].

Whilst a small number of studies, confirmed in a recent meta-analysis[3], suggest osteoporosis as a risk factor in pelvic RRIFs, unlike the strong evidence base for bisphosphonate use in primary and secondary prevention of fragility fractures, there is no such evidence for RRIFs[5]. A small non-controlled study demonstrated intravenous zolendronic acid administration prior to spinal radiotherapy led to a lower prevalence of radiotherapy bone toxicity than expected[10] and a single randomised prospective study in

For peer review only - http://bmjopen.bmj.com/site/about/guidelines.xhtml

patients undergoing spinal radiotherapy for metastatic disease demonstrated that intravenous zolendronic acid reduced urinary markers of collagen cross linking[11].

Contradictory data from animal studies around the protective effects of bisphosphonates on RRIFs limits our understanding of the pathophysiology and therapeutics of RRIFs. Animal studies using whole mouse radiation have demonstrated an early activation of bone resorption in the 5 days following low dose (2 Gy) of radiotherapy which was reduced by subcutaneous administration of risedronate immediately following irradiation[12]. In contrast, a focal radiation technique in mice (using a small animal radiation research platform or SARRP), arguably a more physiological representative method of irradiation, demonstrated that alendronic acid did not prevent the radiation induced trabecular bone loss but that this was prevented by blocking osteoblast apoptosis with PTH 1-34[13].

The RadBone is the first open-label prospective randomised controlled trial (RCT) to determine the feasibility and acceptability of a musculoskeletal health package (MHP) intervention in women undergoing pelvic radiotherapy for gynaecological malignancies and inform power calculations for a definitive RCT. Moreover, this feasibility trial will also explore potential implications on the incidence of RRIFs, quality of life and other clinical effectiveness and safety outcomes, as well as providing indicative estimates of the intervention's cost-effectiveness.

For peer review only - http://bmjopen.bmj.com/site/about/guidelines.xhtml

Methods and analysis:

Study Design (Figure 1)

Study setting

The planned study is a prospective randomised controlled feasibility trial of eighty patients with gynaecological malignancy (cervical and endometrial) undergoing pelvic radiotherapy at the Christie Hospital NHS Foundation Trust in Manchester, UK (a tertiary referral Oncology centre). The study opened for recruitment in May 2021, and the estimated primary completion date is in November 2022 and study completion date in June 2023.

Eligibility Criteria

Individuals aged over 18 years, with a histologically confirmed endometrial or cervical cancer undergoing potentially curative or adjuvant radiotherapy will be eligible, provided they are able and willing to provide an informed consent to participate.

The exclusion criteria are (i) age less than 18 years or greater than 85 years; (ii) pre-existing bone conditions such as osteoporosis treated with bisphosphonates in the previous 5 years, fibrous dysplasia, osteogenesis imperfecta, or other metabolic bone conditions; (iii) home address outside Greater Manchester; (iv) contraindication or intolerance of Magnetic Resonance scanning.

Interventions

Women undergoing radiotherapy for a gynaecological malignancy will be randomised to an observation (Ob) group and will receive standard assessment and care, following the current local clinical pathway, or an intervention group that will receive a “musculoskeletal health package” (MHP), in addition to standard assessment and care and will be followed for 18 months.

Patients randomised to the MHP arm will receive (i) a physical assessment of musculoskeletal health and a 3-month prehabilitation personalised exercise package as part of the Greater Manchester prehab4cancer program[14], (ii) a fracture risk assessment (FRAX) based on baseline dual-energy x-ray absorptiometry (DXA) bone mineral density (BMD), and (iii) treatment for bone health according to national UK recommendations i.e., standard of

For peer review only - http://bmjopen.bmj.com/site/about/guidelines.xhtml

care for prevention of fragility fractures, by being subdivided into 3 groups (low risk, medium risk and high risk).

Patients with a normal BMD and a FRAX score below the National Osteoporosis Guideline Group (NOGG) recommended treatment line will be considered low risk. Medium risk is defined as osteopenia on the DXA, with FRAX score below the NOGG treatment line. Finally, those with osteopenia and a previous vertebral or hip fracture, or a FRAX score above the NOGG recommended treatment line will be considered high risk.

Low risk patients will be provided with a copy of the Royal Osteoporosis Society (ROS) "Healthy living for strong bones" leaflet. In addition to the leaflet, medium risk patients will receive calcium (1000 mg once daily) and vitamin D (800 IU per day) supplementation. The same interventions will be offered to high-risk patients, who will also undergo secondary osteoporosis screening (blood tests) and will receive oral alendronate 70mg once weekly, in the absence of contraindications. Annual intravenous zolendronic acid infusion will be considered as an alternative where appropriate.

Those randomised to the observation arm will remain blinded to the results of the evaluations until the end of the study unless a fragility fracture or RRIF develops during the study.

Prehabilitation Exercise Programme (Prehab4cancer)

All patients randomised to the MHP arm of the study will be offered a bespoke prehabilitation exercise programme via the Prehab4cancer programme in Greater Manchester. The MHP arm patients will be referred to the Prehab4cancer team via electronic referral immediately following randomisation. Allocated patients will be individually assessed by the Prehab4cancer team according to their usual protocols and assigned an appropriate prehabilitation program. Duration of the programme is 12 weeks from the first assessment and participation will be encouraged, as tolerated. The Prehab4Cancer and recovery programme is community-based, which incorporates exercise (cardiovascular and muscle strengthening/resistance training), nutritional screening, and advice and wellbeing support. Further details of programmes' assessment tools and the stratification of interventions are described by Moore et al[14] and can be found here: www.prehab4cancer.co.uk. The current scope of this protocol is to evaluate feasibility of participants' engagement in this face to face and remote prehabilitation service both pre- and during treatment.

Baseline and Follow-up Evaluation (Figure 2)

For peer review only - http://bmjopen.bmj.com/site/about/guidelines.xhtml

As described in figure 2, baseline evaluations will include a bone health assessment with DXA BMD measurement and completion of a bone health questionnaire. PROMs will also be captured. Finally, fasting serum and plasma blood samples will also be collected.

At 6, 12 and 18 months post radiotherapy all patients will undergo a pelvic MRI assessment for RRIFs, PROMs assessment and fasting blood sampling. During the final visit, at 18 months, all patients will have a DXA BMD scan and physical assessment of their musculoskeletal health. If signs or symptoms compatible with a RRIF are described outside the study visits study participants will be assessed and managed following the current clinical pathways.

Imaging studies

DXA scans of the total hip, femoral neck, L1-L4 spine and TBS assessments will be performed on a single DXA scanner (Hologic Horizon A SN 300792M version 5.6.07 with TBS v.3.0.2 calibrated to the above scanner) at the Christie NHS Foundation Trust as per local protocol. These will be undertaken by two technicians trained in conducting DXA. Images will be reviewed, validated and interpreted by the lead investigator (CEH). The femoral neck BMD (g/cm^2) will be used in conjunction with a standardised DXA questionnaire to complete FRAX calculation.

Pelvic Magnetic Resonance Imaging (MRI) scans will be performed at 6, 12 and 18 months on a 1.5T MRI scanner at the Christie Hospital by trained radiographers in accordance with the study imaging protocol. Four pelvic sequences will be performed per patient (5mm slice thickness, field of view 400mm; coronal T1, coronal STIR (inversion time 150ms), axial T1 and axial STIR (inversion time 165 ms). These will correspond to routine follow-up scans where possible. All bone sequence scans will be dual reported by 2 consultant radiologists who will document the presence of fracture and their confidence in its presence, fracture location, fracture line, bone marrow oedema and other abnormalities.

Biochemical studies

Fasting blood tests will be performed at baseline, weekly during radiotherapy (visits 2 to 10, one day prior to chemotherapy if receiving) and at 6, 12 and 18 months in all patients. Patients allocated to the MHP High risk arm and started on oral bisphosphonate therapy will have an additional bone turnover marker blood test at 3 months to assess bisphosphonate efficacy. All samples will be taken simultaneously with routinely collected clinical blood samples where appropriate.

As part of the MHP intervention arm, blood will be sampled, analysed, and assessed at baseline, 6, 12 and 18 months for the measurement of full blood count, urea and electrolytes, liver function tests, parathyroid hormone, vitamin D, thyroid function test,

For peer review only - http://bmjopen.bmj.com/site/about/guidelines.xhtml

oestradiol, HbA1c, procollagen type 1 amino-terminal propeptide (P1NP), and the beta-C-terminal telopeptide (CTx). Moreover, in the observation arm, serum samples will be collected, processed and stored at -80°C for batch analysis at the end of the study.

Additional fasting blood samples will be collected at all timepoints mentioned for longitudinal analysis of bone turnover markers and for proteomic analysis. These samples will be processed and stored at -80°C, following local standard operating procedures (SOPs), for batch analysis at the end of the study. Bone turnover markers will be evaluated using ELISA techniques and will include CTX, NTX, P1NP, osteocalcin, TRAcP5b and bone ALP.

Sequential Window Acquisition of all Theoretical Mass Spectra (SWATH-MS)

Proteomic analysis will be conducted at the Stoller Biomarker Discovery Centre, following local SOPs[15]. Samples will be analysed by a Data Independent Acquisition method known as SWATH-MS with a micro-flow LC-MS system comprising an Eksigent nanoLC 400 autosampler and an Eksigent nanoLC pump coupled to a SCIEX 6600 Triple-TOF mass spectrometer (68 min run-time). When SWATH maps are generated, the presence and abundance of plasma proteins will be quantified using published plasma reference libraries. Differential expression analysis will be used to identify candidate biomarkers using artificial intelligence approaches. Linear regression will be used to detect correlations with the presence of RRIFs and BMD.

Few longitudinal studies have tracked proteins of interest over the whole course of radiotherapy from pre-treatment baseline through to follow-up. We have undertaken one pilot that shows the potential value of this work[16]. Other studies that have investigated this have demonstrated distinguishing profiles with groups of approximately n=30. Two pre-radiotherapy baseline samples will be used to assess natural variation and comparison with the variance of measurements following radiotherapy and further comparison between the MHP and observation arm (n=40 per group).

Electronic data will be pseudoanonymised (coded) to protect the identity of the participants.

PROMS and Health Utilisation Proforma

PROMs will be collected either as electronic PROMS (using the myChristie, myHealth application) or paper-based PROMS at baseline 6, 12, and 18 months.

The evaluated PROMs will include the adapted pelvic patient-reported outcome version of the common terminology criteria for adverse events (PRO-CTCAE) assessment, the Short

For peer review only - http://bmjopen.bmj.com/site/about/guidelines.xhtml

Musculoskeletal Function Assessment (SMFA) modified for lower limb, the 5-level version of the EuroQol tool (EQ-5D-5L) and a tailored Health Utilisation Proforma.

The CTCAE pelvic questionnaire will include as measures bowel questions scored out of 22, urinary questions out of 19 and sexual questions out of 8, with a total out of 49; a higher score indicates worse quality of life. The adapted SMFA questionnaire includes 39 questions, with a minimum possible score of 39 and maximum of 195; scores are standardised with high scores indicating poor function.

Criteria for discontinuing

Participants may decide to withdraw from the study at any time. Discontinuation of the study participants may occur as a result of investigator decision, safety concerns, and significant non-compliance to the protocol or incorrect enrolment. Reasons for discontinuation will be captured.

As this is a feasibility study, participants may decide to discontinue their participation in certain aspects of the study (for example declining the prehabilitation programme or deciding not to take recommended medications). The participants can continue with the study and the details will be captured in the case report form (CRF).

Outcomes

The primary outcomes for this feasibility study will inform the design and power calculations for a definitive UK multi-centre RCT. These are:

1) Eligibility and screening rate: proportion of patients eligible for the study from patient population. [Assessed at baseline]
2) Recruitment and study group allocation rate: number and proportion of eligible patients recruited, randomised and allocated to appropriate study populations. [Assessed 2 weeks post consent]
3) Intervention fidelity rate: number and proportion of patients completing the elements of the study (assessment visits, prehab exercise programme, prescribed medications, QOL questionnaire). [Assessed at the end of study, at 18 months]
4) Attrition rate: number of patients lost to follow-up. [Assessed at the end of study, at 18 months]
5) Patient and physician acceptability assessed with electronic questionnaires. [Change from baseline assessed at 6,12 and 18 months]
6) Health Economic Analysis: within-trial cost-effectiveness analysis to demonstrate feasibility of health economic data collection and analysis in a multi-centre RCT. [Change from baseline assessed at 6,12 and 18 months]

The secondary outcomes are:

1) Incidence of pelvic Radiotherapy Related Insufficiency Fracture (RRIF). [Assessed at 6, 12 and 18 months post radiotherapy]

For peer review only - http://bmjopen.bmj.com/site/about/guidelines.xhtml

2) Longitudinal change in BMD and fracture risk using FRAX. [Assessed at baseline and 18 months]
3) Longitudinal change in biochemical markers of bone turnover. [Change from baseline assessed at 2, 3, 4, 5, 6, 7, 8 and 9 weeks and at 6, 12 and 18 months]
4) Quality of Life assessment: adapted CTCAE pelvic questionnaire and SMFA adapted to lower limbs. [Change from baseline assessed at 6, 12 and 18 months]

Exploratory Endpoints include identification of predictive markers of RRIFs (radiomic, proteomic, BMD) and exploratory measurement of proteomic biomarkers of bone turnover during pelvic radiotherapy.

Sample Size

No formal power calculation has been performed as this is a feasibility study. The study will collect initial data such as measures of location and variability for key outcome measures. It is recognised that in general, 30 patients are required in order to estimate such parameters[17]. For this study a total of 80 patients will be recruited and randomised with equal probability to either the MHP or observation arms (i.e. 40 per group). Assuming attrition rates of 15% per group, at least 30 should remain in each arm. This should be sufficient to assess the feasibility of a larger RCT study and estimate group means, standard deviations and percentages for key outcomes.

Recruitment

80 patients will be recruited over an 18-month period, approximately 4 patients per month. As this is a feasibility study there will be no interim analysis of study results.

Assignment of interventions

Consenting, eligible participants will be randomised to the MHP or observation group using a validated online service; sealedenvelope™ (https://www.sealedenvelope.com). A permuted block (block size:4) randomisation protocol will be utilised with a 1:1 allocation (MHP to observation arm).

Data Collection, management and analysis

Statistical and Health Economic Analysis

As this is a feasibility study, it will not involve hypothesis testing to identify whether the intervention has had an impact. Instead, data analysis will be descriptive, focusing on the percentage of patients in each group developing RRIFs and risk factors for this. Means and a measure of variation will be calculated for each secondary outcome. These data, along with

For peer review only - http://bmjopen.bmj.com/site/about/guidelines.xhtml

estimates of recruitment and attrition rates, will help inform a power calculation for the definitive trial.

A within-trial cost-effectiveness analysis[18] will be undertaken from the perspective of the UK National Health Service (NHS). Cost data for the intervention arm will reflect resource use associated with the musculoskeletal health package and treatment costs for both the control and intervention arm will be taken into account. Resource use will be extracted from patient records and the health care utilisation proforma. Relevant sources (e.g., NHS reference costs) will be used to identify unit costs. Health related quality of life (HRQL) scores will be generated using the EQ-5D-5L at baseline and at each of the three follow-up time points (6, 12, 18 months).

A descriptive analysis of the costs and outcomes data will be completed focusing on: a. whether the EQ-5D-5L and SMFA are able to adequately capture differences in health status before and after implementation of the musculoskeletal health package and across both treatment arms of the study; b. whether the resource-use survey is able to record data necessary to enable a full cost-effectiveness analysis; c. the nature of missing data for the EQ-5D-5L, SMFA and resource-use survey to assess responses, sensitivity, and any patterns within the missing data.

A within-trial cost-effectiveness analysis will be conducted to provide an indicative estimate of cost-effectiveness. Between-arm differences in costs and outcomes will be expressed as an incremental cost-effectiveness ratio (ICERs): the cost per QALY gained from the intervention compared to usual care. ICERs will also be calculated using the SMFA in an additional scenario analysis.

Trial oversight

An internal trial management group will be convened for the study, consisting of the chief investigator, project manager, Clinical Trials administrator, research nurse and a representative of the research and innovation division (R+I) as core members. The group will meet monthly. The study sponsor (Christie Hospital NHS Foundation Trust) will monitor the conduct of the trial.

Patient and Public Involvement

This protocol was developed with the participation of the Christie pelvic radiotherapy user group and supported by the Pelvic Radiation Disease Association (PRDA).

Ethics and Dissemination

For peer review only

For peer review only - http://bmjopen.bmj.com/site/about/guidelines.xhtml

This study has been approved by GM East Research Ethics Committee in November 2020 (REC reference 20/NW/0410) and is registered at clinicaltrials.gov (ClinicalTrials.gov Identifier: NCT04555317). The study opened for recruitment in May 2021. The results of this study will be published in peer reviewed journals, will be presented in national and international conferences, and will be communicated to relevant stakeholders. Moreover, a plain English report will be shared with the study participants, patients' organizations, and media.

Data sharing statement

Consent to share data from this study for future research is voluntary. To ensure compliance with regulatory and governance requirements, approval from the sponsor team is required prior to the release of any data generated by this Christie sponsored study, to a third party. Any requests are to be directed towards the-christie.sponsoredresearch@nhs.net for consideration and must follow all local Policies and review procedures. If a proposal is accepted, then the sponsor will work with the requestor to develop any necessary data transfer plans/agreements.

Authors' contributions: CEH developed the protocol, MRC application and ethics application for the study. RB is the MRC-CARP academic partner to CEH and contributed to the study design and protocol.

KJ, LHB, KH contributed to the development of the gynae-oncology aspects

RK, SO'C, TW contributed to the development of the MRI radiology aspects

ZM, JM contributed to the development of the Prehab4Cancer aspects

ST, JY contributed to the PROMS development

ME contributed to the Health Economic Analysis

ADW, IBJ contributed to the proteomic and data analysis

VCG and CEH prepared the manuscript

All authors: critically reviewed and commented on the manuscript.

Funding statement: This work is partially supported by an MRC-NIHR Clinical Academic Research Partnership) award: grant number MR/T024887/1. Funding is being sought for Health Economic Analysis, QOL and PROMS development and longitudinal proteomic analysis. Equipment used in the Stoller Biomarker Discovery Centre is funded by a donation received from the Stoller Charitable Trust and a research grant awarded by the Medical

For peer review only

For peer review only - http://bmjopen.bmj.com/site/about/guidelines.xhtml

448 Research Council (MR/M008959/1). MRC/EPSRC Molecular Pathology Node provided 449 additional financial support by a grant from the Medical Research Council and Engineering & 450 Physical Sciences Research Council (MR/N00583X/1).

451 **Competing interests statement.** No competing interests

452

For peer review only

Figures legends

Figure 1: Recruitment, randomisation process and description of the stratified interventions. (#: fracture)

Figure 2: Study flow chart; assessments and outcome time-points.

C+R = Consent and Randomisation, MHP= Musculoskeletal Health Package arm, Ob = observational arm, HR = high risk, CV = clinic visit, EBRT= External Beam Radiotherapy. NHS: National Health System

For peer review only

For peer review only - http://bmjopen.bmj.com/site/about/guidelines.xhtml

Full references

1. Available from: https://www.macmillan.org.uk/_images/cancer-statistics-factsheet_tcm9-260514.pdf [Accessed February 2022]
2. Higham CE, Faithfull S. Bone Health and Pelvic Radiotherapy. Clin Oncol (R Coll Radiol). 2015;27(11):668-678. doi:10.1016/j.clon.2015.07.006
3. Sapienza LG, Salcedo MP, Ning MS, et al. Pelvic Insufficiency Fractures After External Beam Radiation Therapy for Gynecologic Cancers: A Meta-analysis and Meta-regression of 3929 Patients. Int J Radiat Oncol Biol Phys. 2020;106(3):475-484. doi:10.1016/j.ijrobp.2019.09.012
4. Gebauer J, Higham C, Langer T, Denzer C, Brabant G. Long-Term Endocrine and Metabolic Consequences of Cancer Treatment: A Systematic Review. Endocr Rev. 2019;40(3):711-767. doi:10.1210/er.2018-00092
5. van den Blink QU, Garcez K, Henson CC, Davidson SE, Higham CE. Pharmacological interventions for the prevention of insufficiency fractures and avascular necrosis associated with pelvic radiotherapy in adults. Cochrane Database Syst Rev. 2018;4(4):CD010604. Published 2018 Apr 23. doi:10.1002/14651858.CD010604.pub2.
6. Center JR, Nguyen TV, Schneider D, Sambrook PN, Eisman JA. Mortality after all major types of osteoporotic fracture in men and women: an observational study. Lancet. 1999;353(9156):878-882. doi:10.1016/S0140-6736(98)09075-8
7. Taillandier J, Langue F, Alemanni M, Taillandier-Heriche E. Mortality and functional outcomes of pelvic insufficiency fractures in older patients. Joint Bone Spine. 2003;70(4):287-289. doi:10.1016/s1297-319x(03)00015-0
8. Oh D, Huh SJ, Nam H, et al. Pelvic insufficiency fracture after pelvic radiotherapy for cervical cancer: analysis of risk factors. Int J Radiat Oncol Biol Phys. 2008;70(4):1183-1188. doi:10.1016/j.ijrobp.2007.08.005
9. Holch P, Pini S, Henry AM, et al. eRAPID electronic patient self-Reporting of Adverse-events: Patient Information and aDvice: a pilot study protocol in pelvic radiotherapy. Pilot Feasibility Stud. 2018;4:110. Published 2018 Jun 5. doi:10.1186/s40814-018-0304-6
10. Pichon B, Campion L, Delpon G, et al. High-Dose Hypofractionated Radiation Therapy for Noncompressive Vertebral Metastases in Combination With Zoledronate: A Phase 1 Study. Int J Radiat Oncol Biol Phys. 2016;96(4):840-847. doi:10.1016/j.ijrobp.2016.07.027
11. Gierloff M, Reutemann M, Gülses A, Niehoff P, Wiltfang J, Açil Y. Effects of zoledronate on the radiation-induced collagen breakdown: a prospective randomized clinical trial. Clin Transl Oncol. 2015;17(6):454-461. doi:10.1007/s12094-014-1257-8
12. Willey JS, Livingston EW, Robbins ME, et al. Risedronate prevents early radiation-induced osteoporosis in mice at multiple skeletal locations. Bone. 2010;46(1):101-111. doi:10.1016/j.bone.2009.09.002
13. Chandra A, Lin T, Tribble MB, et al. PTH1-34 alleviates radiotherapy-induced local bone loss by improving osteoblast and osteocyte survival. Bone. 2014;67:33-40. doi:10.1016/j.bone.2014.06.030
14. Moore J, Merchant Z, Rowlinson K, et al. Implementing a system-wide cancer prehabilitation programme: The journey of Greater Manchester's 'Prehab4cancer'. Eur J Surg Oncol. 2021;47(3 Pt A):524-532. doi:10.1016/j.ejso.2020.04.042
15. Geary B, Walker MJ, Snow JT, et al. Identification of a Biomarker Panel for Early Detection of Lung Cancer Patients. J Proteome Res. 2019;18(9):3369-3382. doi:10.1021/acs.jproteome.9b00287
16. Walker MJ, Zhou C, Backen A, et al. Discovery and Validation of Predictive Biomarkers of Survival for Non-small Cell Lung Cancer Patients Undergoing Radical Radiotherapy: Two Proteins With Predictive Value. EBioMedicine. 2015;2(8):841-850. Published 2015 Jun 19. doi:10.1016/j.ebiom.2015.06.013
17. Billingham SA, Whitehead AL, Julious SA. An audit of sample sizes for pilot and feasibility trials being undertaken in the United Kingdom registered in the United Kingdom Clinical Research Network database. BMC Med Res Methodol. 2013;13:104. Published 2013 Aug 20. doi:10.1186/1471-2288-13-104
18. Drummond MF, Sculpher MJ, Claxton K, Stoddart GL, Torrance GW. Methods for the Economic Evaluation of Health Care Programmes. 4th ed. Oxford: Oxford University Press, Oxford. <https://books.google.co.uk/books?id=lvWACgAAQBAJ>

For peer review only

For peer review only - http://bmjopen.bmj.com/site/about/guidelines.xhtml

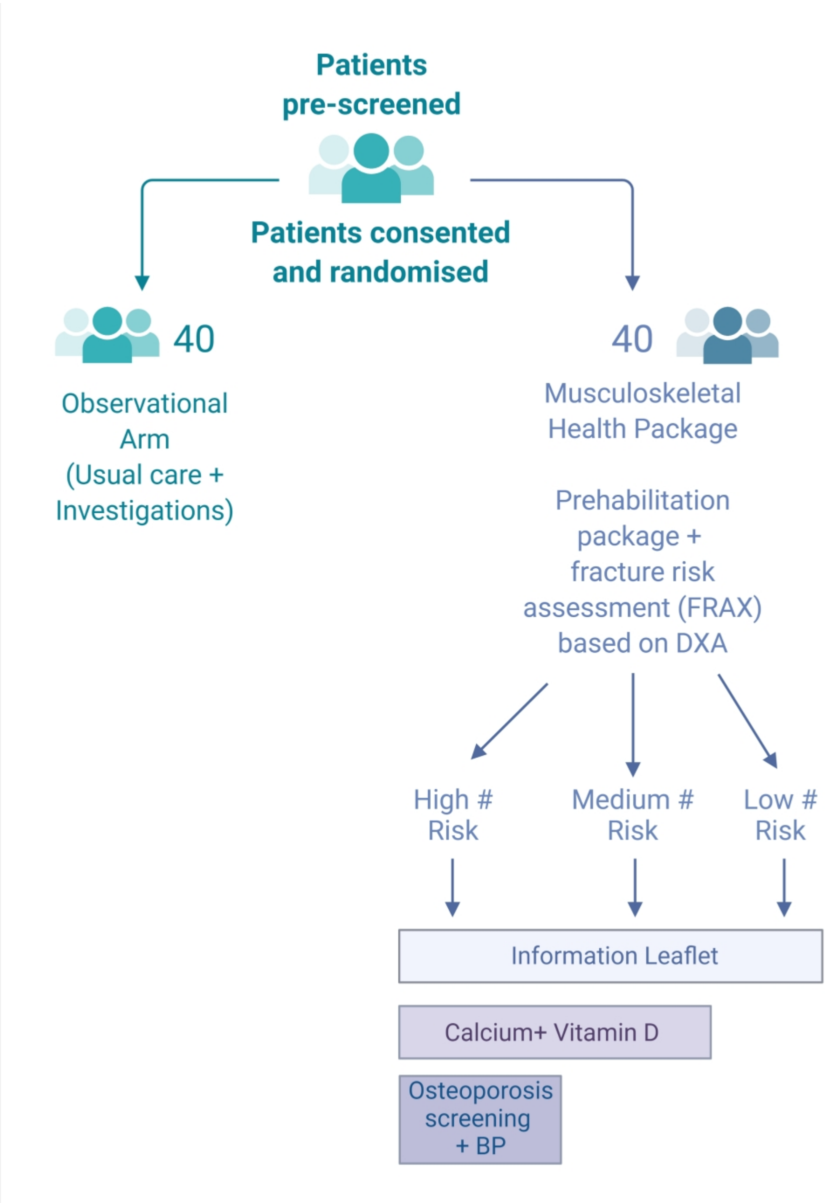

Figure 1: Recruitment, randomisation process and description of the stratified interventions. (#: fracture)

218x310mm (300 x 300 DPI)

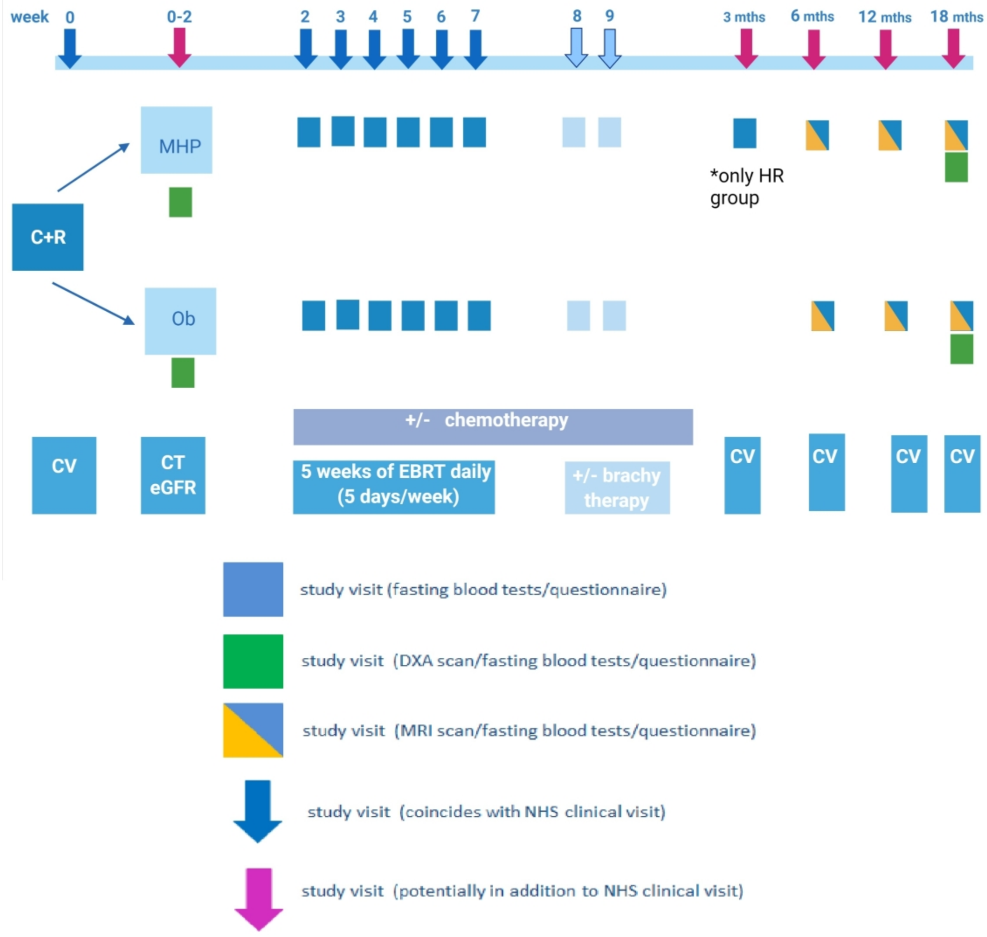

Figure 2: Study flow chart; assessments and outcome time-points.
C+R = Consent and Randomisation, MHP= Musculoskeletal Health Package arm, Ob = observational arm, HR = high risk, CV = clinic visit, EBRT= External Beam Radiotherapy. NHS: National Health System

257x246mm (300 x 300 DPI)

Reporting checklist for protocol of a clinical trial.

Based on the SPIRIT guidelines.

Instructions to authors

Complete this checklist by entering the page numbers from your manuscript where readers will find each of the items listed below.

Your article may not currently address all the items on the checklist. Please modify your text to include the missing information. If you are certain that an item does not apply, please write "n/a" and provide a short explanation.

Upload your completed checklist as an extra file when you submit to a journal.

In your methods section, say that you used the SPIRITreporting guidelines, and cite them as:

Chan A-W, Tetzlaff JM, Gøtzsche PC, Altman DG, Mann H, Berlin J, Dickersin K, Hróbjartsson A, Schulz KF, Parulekar WR, Krleža-Jerić K, Laupacis A, Moher D. SPIRIT 2013 Explanation and Elaboration: Guidance for protocols of clinical trials. BMJ. 2013;346:e7586

		Reporting Item	Page Number
**Administrative information**			
Title	#1	Descriptive title identifying the study design, population, interventions, and, if applicable, trial acronym	1
Trial registration	#2a	Trial identifier and registry name. If not yet registered,	2

For peer review only

		name of intended registry	
Trial registration: data set	#2b	All items from the World Health Organization Trial Registration Data Set	6-14
Protocol version	#3	Date and version identifier	N/A
Funding	#4	Sources and types of financial, material, and other support	14,15
Roles and responsibilities: contributorship	#5a	Names, affiliations, and roles of protocol contributors	14
Roles and responsibilities: sponsor contact information	#5b	Name and contact information for the trial sponsor	13,14
Roles and responsibilities: sponsor and funder	#5c	Role of study sponsor and funders, if any, in study design; collection, management, analysis, and interpretation of data; writing of the report; and the decision to submit the report for publication, including whether they will have ultimate authority over any of these activities	13,14
Roles and responsibilities: committees	#5d	Composition, roles, and responsibilities of the coordinating centre, steering committee, endpoint adjudication committee, data management team, and other individuals or groups overseeing the trial, if applicable (see Item 21a for data monitoring committee)	13,14

Introduction

For peer review only

Background and rationale	#6a	Description of research question and justification for undertaking the trial, including summary of relevant studies (published and unpublished) examining benefits and harms for each intervention	5
Background and rationale: choice of comparators	#6b	Explanation for choice of comparators	5
Objectives	#7	Specific objectives or hypotheses	4,5
Trial design	#8	Description of trial design including type of trial (eg, parallel group, crossover, factorial, single group), allocation ratio, and framework (eg, superiority, equivalence, non-inferiority, exploratory)	5
**Methods: Participants, interventions, and outcomes**			
Study setting	#9	Description of study settings (eg, community clinic, academic hospital) and list of countries where data will be collected. Reference to where list of study sites can be obtained	6
Eligibility criteria	#10	Inclusion and exclusion criteria for participants. If applicable, eligibility criteria for study centres and individuals who will perform the interventions (eg,	7

For peer review only

		surgeons, psychotherapists)	
Interventions: description	#11a	Interventions for each group with sufficient detail to allow replication, including how and when they will be administered	7,8
Interventions: modifications	#11b	Criteria for discontinuing or modifying allocated interventions for a given trial participant (eg, drug dose change in response to harms, participant request, or improving / worsening disease)	11
Interventions: adherance	#11c	Strategies to improve adherence to intervention protocols, and any procedures for monitoring adherence (eg, drug tablet return; laboratory tests)	N/A
Interventions: concomitant care	#11d	Relevant concomitant care and interventions that are permitted or prohibited during the trial	7,8
Outcomes	#12	Primary, secondary, and other outcomes, including the specific measurement variable (eg, systolic blood pressure), analysis metric (eg, change from baseline, final value, time to event), method of aggregation (eg, median, proportion), and time point for each outcome. Explanation of the clinical relevance of chosen efficacy and harm outcomes is strongly recommended	11,12
Participant timeline	#13	Time schedule of enrolment, interventions (including any run-ins and washouts), assessments, and visits for participants. A schematic diagram is highly recommended (see Figure)	8-11

For peer review only

Sample size	#14	Estimated number of participants needed to achieve study objectives and how it was determined, including clinical and statistical assumptions supporting any sample size calculations	12
Recruitment	#15	Strategies for achieving adequate participant enrolment to reach target sample size	12
**Methods: Assignment of interventions (for controlled trials)**			
Allocation: sequence generation	#16a	Method of generating the allocation sequence (eg, computer-generated random numbers), and list of any factors for stratification. To reduce predictability of a random sequence, details of any planned restriction (eg, blocking) should be provided in a separate document that is unavailable to those who enrol participants or assign interventions	12,13
Allocation concealment mechanism	#16b	Mechanism of implementing the allocation sequence (eg, central telephone; sequentially numbered, opaque, sealed envelopes), describing any steps to conceal the sequence until interventions are assigned	12,13
Allocation: implementation	#16c	Who will generate the allocation sequence, who will enrol participants, and who will assign participants to interventions	12,13

For peer review only

Blinding (masking)	#17a	Who will be blinded after assignment to interventions (eg, trial participants, care providers, outcome assessors, data analysts), and how	5
Blinding (masking): emergency unblinding	#17b	If blinded, circumstances under which unblinding is permissible, and procedure for revealing a participant's allocated intervention during the trial	N/a
**Methods: Data collection, management, and analysis**			
Data collection plan	#18a	Plans for assessment and collection of outcome, baseline, and other trial data, including any related processes to promote data quality (eg, duplicate measurements, training of assessors) and a description of study instruments (eg, questionnaires, laboratory tests) along with their reliability and validity, if known. Reference to where data collection forms can be found, if not in the protocol	9-11
Data collection plan: retention	#18b	Plans to promote participant retention and complete follow-up, including list of any outcome data to be collected for participants who discontinue or deviate from intervention protocols	11
Data management	#19	Plans for data entry, coding, security, and storage, including any related processes to promote data quality (eg, double data entry; range checks for data values).	13

For peer review only

		Reference to where details of data management procedures can be found, if not in the protocol	
Statistics: outcomes	#20a	Statistical methods for analysing primary and secondary outcomes. Reference to where other details of the statistical analysis plan can be found, if not in the protocol	13
Statistics: additional analyses	#20b	Methods for any additional analyses (eg, subgroup and adjusted analyses)	13
Statistics: analysis population and missing data	#20c	Definition of analysis population relating to protocol non-adherence (eg, as randomised analysis), and any statistical methods to handle missing data (eg, multiple imputation)	13
**Methods: Monitoring**			
Data monitoring: formal committee	#21a	Composition of data monitoring committee (DMC); summary of its role and reporting structure; statement of whether it is independent from the sponsor and competing interests; and reference to where further details about its charter can be found, if not in the protocol. Alternatively, an explanation of why a DMC is not needed	13,14
Data monitoring: interim analysis	#21b	Description of any interim analyses and stopping guidelines, including who will have access to these interim results and make the final decision to terminate the trial	12
Harms	#22	Plans for collecting, assessing, reporting, and managing solicited and spontaneously reported adverse events and other unintended effects of trial interventions or trial	11

For peer review only

		conduct	
Auditing	#23	Frequency and procedures for auditing trial conduct, if any, and whether the process will be independent from investigators and the sponsor	14
**Ethics and dissemination**			
Research ethics approval	#24	Plans for seeking research ethics committee / institutional review board (REC / IRB) approval	2
Protocol amendments	#25	Plans for communicating important protocol modifications (eg, changes to eligibility criteria, outcomes, analyses) to relevant parties (eg, investigators, REC / IRBs, trial participants, trial registries, journals, regulators)	13,14
Consent or assent	#26a	Who will obtain informed consent or assent from potential trial participants or authorised surrogates, and how (see Item 32)	7
Consent or assent: ancillary studies	#26b	Additional consent provisions for collection and use of participant data and biological specimens in ancillary studies, if applicable	N/A
Confidentiality	#27	How personal information about potential and enrolled participants will be collected, shared, and maintained in order to protect confidentiality before, during, and after the trial	13
Declaration of	#28	Financial and other competing interests for principal	14,15

For peer review only

interests		investigators for the overall trial and each study site	
Data access	#29	Statement of who will have access to the final trial dataset, and disclosure of contractual agreements that limit such access for investigators	TBC
Ancillary and post trial care	#30	Provisions, if any, for ancillary and post-trial care, and for compensation to those who suffer harm from trial participation	N/A
Dissemination policy: trial results	#31a	Plans for investigators and sponsor to communicate trial results to participants, healthcare professionals, the public, and other relevant groups (eg, via publication, reporting in results databases, or other data sharing arrangements), including any publication restrictions	14
Dissemination policy: authorship	#31b	Authorship eligibility guidelines and any intended use of professional writers	14
Dissemination policy: reproducible research	#31c	Plans, if any, for granting public access to the full protocol, participant-level dataset, and statistical code	14
**Appendices**			
Informed consent materials	#32	Model consent form and other related documentation given to participants and authorised surrogates	TBC
Biological specimens	#33	Plans for collection, laboratory evaluation, and storage of biological specimens for genetic or molecular analysis in the current trial and for future use in ancillary studies, if applicable	N/A

For peer review only

None The SPIRIT Explanation and Elaboration paper is distributed under the terms of the Creative Commons Attribution License CC-BY-NC. This checklist can be completed online using https://www.goodreports.org/, a tool made by the EQUATOR Network in collaboration with Penelope.ai

For peer review only

BMJ Open

RadBone: Bone Toxicity following Pelvic Radiotherapy. A prospective randomised controlled feasibility study evaluating a musculoskeletal health package in women with gynaecological cancers undergoing pelvic radiotherapy.

Journal:	*BMJ Open*
Manuscript ID	bmjopen-2021-056600.R2
Article Type:	Protocol
Date Submitted by the Author:	27-Apr-2022
Complete List of Authors:	Chatzimavridou Grigoriadou, Victoria; Christie Hospital NHS Foundation Trust, Department of Endocrinology; University of Manchester, Manchester Academic Health Science Centre<br>Barraclough, Lisa H; Christie Hospital NHS Foundation Trust, Department of Clinical Oncology<br>Baricevic-Jones, Ivona; Manchester Academic Health Science Centre, Stoller Biomarker Discovery Centre, Division of Cancer Sciences, School of Medical Sciences, Faculty of Biology, Medicine and Health, University of Manchester<br>Bristow, Robert; University of Manchester, Manchester Cancer Research Centre<br>Eden, Martin; The University of Manchester Manchester Centre for Health Economics<br>Haslett, Kate; Christie Hospital NHS Foundation Trust, Department of Clinical Oncology<br>Johnson, Karen; Christie Hospital NHS Foundation Trust, Department of Clinical Oncology<br>Kochhar, Rohit; The Christie National Health Service Foundation Trust, Department of Radiology<br>Merchant, Zoe; Programme Lead for the Greater Manchester ‘Prehab4Cancer and Recovery programme’/Highly Specialist Occupational Therapist. GM Cancer alliance hosted by the Christie NHS Foundation Trust.<br>Moore, John; GM Cancer Clinical Director for Prehabilitation and Recovery University of Manchester and Manchester Metropolitan University; Consultant in Anaesthetics and Intensive Care Medicine, Manchester University NHS Foundation Trust<br>O’Connell, Sarah; The Christie National Health Service Foundation Trust, Department of Radiology<br>Taylor, Sally; The Christie School of Oncology, The Christie Patient Centred Research Team<br>Westwood, Thomas; The Christie National Health Service Foundation Trust, Department of Radiology<br>Whetton, Anthony; University of Manchester, Stoller Biomarker Discovery Centre; University of Manchester, Division of Cancer Sciences, Faculty of Biology, Medicine and Health<br>Yorke, Janelle; The Christie NHS Foundation Trust, Clinical Oncology; The University of Manchester School of Health Sciences, Christie Patient-

	Centred Research, Division of Nursing, Midwifery & Social Work<br>Higham, Claire E; Christie Hospital NHS Foundation Trust, Department of Endocrinology; The University of Manchester, Manchester Academic Health Science Centre
**Primary Subject Heading**:	Diabetes and endocrinology
Secondary Subject Heading:	Oncology
Keywords:	RADIOTHERAPY, Musculoskeletal disorders < ORTHOPAEDIC & TRAUMA SURGERY, Gynaecological oncology < GYNAECOLOGY, Clinical trials < THERAPEUTICS

SCHOLARONE™
Manuscripts

For peer review only - http://bmjopen.bmj.com/site/about/guidelines.xhtml

Title: RadBone: Bone Toxicity following Pelvic Radiotherapy. A prospective randomised controlled feasibility study evaluating a musculoskeletal health package in women with gynaecological cancers undergoing pelvic radiotherapy.

Authors:

Victoria Chatzimavridou-Grigoriadou[1], Lisa H Barraclough[2], Ivona Baricevic-Jones[3], Robert G Bristow[4], Martin Eden[5], Kate Haslett[2], Karen Johnson[2], Rohit Kochhar[6], Zoe Merchant[7], John Moore[8], Sarah O'Connell[6], Sally Taylor[9], Thomas Westwood[6], Anthony D Whetton[3], Janelle Yorke[9], Claire E Higham[1]

Affiliations:

[1]Department of Endocrinology, Christie Hospital NHS Foundation Trust, Manchester, UK; University of Manchester, Manchester Academic Health Science Centre, Manchester, UK

[2]Department of Clinical Oncology, Christie Hospital NHS Foundation Trust, Manchester, UK.

[3] Stoller Biomarker Discovery Centre, Division of Cancer Sciences, School of Medical Sciences, Faculty of Biology, Medicine and Health, University of Manchester, Manchester Academic Health Science Centre, Manchester, UK.

[4]Division of Cancer Sciences, The University of Manchester, Manchester, UK; CRUK Manchester Institute and Manchester Cancer Research Centre, Manchester, UK.

[5]Manchester Centre for Health Economics, The University of Manchester, Oxford Road, M13 9PL

[6]Department of Radiology, The Christie National Health Service Foundation Trust, Manchester, United Kingdom.

[7]Programme Lead for the Greater Manchester 'Prehab4Cancer and Recovery programme'/Highly Specialist Occupational Therapist. GM Cancer alliance hosted by the Christie NHS Foundation Trust.

[8]Consultant in Anaesthetics and Intensive Care Medicine, Manchester University NHS Foundation Trust. GM Cancer Clinical Director for Prehabilitation and Recovery. University of Manchester and Manchester Metropolitan University.

[9]The Christie Patient Centred Research Team, The Christie School of Oncology, The Christie NHS Foundation Trust, Wilmslow Road, Manchester, UK.

For peer review only

Corresponding authors:

- Dr Claire E Higham, DPhil, FRCP, Consultant Endocrinologist, Department of Endocrinology, Christie Hospital NHS Foundation Trust, Manchester, UK; University of Manchester, Manchester Academic Health Science Centre, Manchester, UK. claire.higham2@nhs.net
- Dr Victoria Chatzimavridou-Grigoriadou, MBBS, MRCP (UK), NIHR Academic Clinical Fellow in Endocrinology and Diabetes, Department of Endocrinology, Christie Hospital NHS Foundation Trust, Manchester, UK; University of Manchester, Manchester Academic Health Science Centre, Manchester, UK. victoriachgr@gmail.com

Abstract:

Introduction: Patients receiving radiotherapy are at risk of developing Radiotherapy Related Insufficiency Fractures (RRIFs), which are associated with increased morbidity and pose a significant burden to patients' quality of life and to the health system. Therefore, effective preventive techniques are urgently required. The RadBone randomized controlled trial (RCT) aims to determine the feasibility and acceptability of a musculoskeletal health package (MHP) intervention in women undergoing pelvic radiotherapy for gynaecological malignancies and to preliminary explore clinical effectiveness of the intervention.

Methods and Analysis: The RadBone RCT will evaluate the addition to standard care of a MHP consisting of a physical assessment of the musculoskeletal health, a three-month prehabilitation personalised exercise package, as well as an evaluation of the fracture risk and if required the prescription of appropriate bone treatment including calcium, vitamin D and -for high-risk individuals- bisphosphonates. Forty participants will be randomized in each group (MHP or observation) and will be followed for 18 months. The primary outcome of this RCT will be feasibility, including the eligibility, screening and recruitment rate, intervention fidelity and attrition rates; acceptability; and health economics. Clinical effectiveness and bone turnover markers will be evaluated as secondary outcomes.

Ethics and dissemination: This study has been approved by the Greater Manchester East Research Ethics Committee (Reference: 20/NW/0410, November 2020). The results will be published in peer reviewed journals, will be presented in national and international conferences, and will be communicated to relevant stakeholders. Moreover, a plain English report will be shared with the study participants, patients' organizations, and media.

Clinical trial registration: NCT04555317.

For peer review only

For peer review only - http://bmjopen.bmj.com/site/about/guidelines.xhtml

Keywords: radiotherapy, insufficiency fractures, musculoskeletal health, gynaecological cancer, randomised control trial.

Strengths and Limitations of this study

- The RadBone is the first randomized controlled trial to assess a musculoskeletal health package aimed to prevent radiotherapy related insufficiency fractures (RRIFs).
- A feasibility economic evaluation will allow future assessment of this complex intervention's cost-effectiveness.
- Planned longitudinal proteomic analyses may reveal mechanistic insights and promising treatment targets.
- A prospectively published detailed protocol increases the transparency and allows for peer review of the methodology used.
- This study is not blinded and lacks an active comparator, hence, it is susceptible to performance and detection bias.

Word count: 3,740

For peer review only

For peer review only - http://bmjopen.bmj.com/site/about/guidelines.xhtml

Introduction

In 2015 there were 2.5 million people in the UK with a diagnosis of cancer and this number is expected to rise to 4 million by 2030[1]. As a result of the continuing improvement in early detection of disease and improved treatment efficacy, a significant proportion are living long beyond their cancer diagnosis. However, estimates suggest that currently over 500,000 people living with and beyond cancer have one or more physical or psychosocial consequences of their cancer or its treatment that affect their lives on a long-term basis. These consequences also have a substantial implication in terms of NHS resources.

Patients receiving radiotherapy are at risk of developing radiotherapy related bone toxicity, in particular radiotherapy related insufficiency fractures (RRIFs). Incidence of RRIFs following pelvic radiotherapy has been reported as between 1.7 and 89% and occurring between 3 to 20 months post radiotherapy. The wide variation in reported incidence depends on imaging modality and radiological reporting standards, symptomatic versus asymptomatic fractures, radiotherapy dose and underlying tumour type (reviewed in[2]). A recent meta-analysis of over 400 patients with RRIFs following pelvic radiotherapy for gynaecological cancers suggested an overall incidence of 14%[3]. Over 30 studies have been published since the 1990's describing more than 1000 patients with pelvic RRIFs. This literature is notable for being almost exclusively retrospective in nature, a sparsity of baseline assessment of bone density and fracture risk, the absence of Patient Reported Outcome Measures (PROMs) used to assess Quality of Life (QOL) and no primary preventative or secondary management intervention studies[4,5].

The devastating effects of osteoporotic fragility fractures on morbidity and mortality and the economic cost are well described[6]. Pelvic insufficiency fractures may also increase mortality [7] but these data reflect an elderly population with multiple co-morbidities and the applicability to the pelvic radiotherapy population is not well defined. In addition, there are no pelvic RRIF studies reporting QOL as an outcome measure. However, the anxiety, pain, reduced mobility and increased morbidity associated with these has been described, with a number of patients requiring hospital admission for assessment and pain control[8]. Therefore, formal studies of QOL and PROMs are much needed, considering the wide range of pelvic radiotherapy toxicities[9].

Whilst a small number of studies, confirmed in a recent meta-analysis[3], suggest osteoporosis as a risk factor in pelvic RRIFs, unlike the strong evidence base for bisphosphonate use in primary and secondary prevention of fragility fractures, there is no such evidence for RRIFs[5]. A small non-controlled study demonstrated intravenous zolendronic acid administration prior to spinal radiotherapy led to a lower prevalence of radiotherapy bone toxicity than expected[10] and a single randomised prospective study in

For peer review only - http://bmjopen.bmj.com/site/about/guidelines.xhtml

patients undergoing spinal radiotherapy for metastatic disease demonstrated that intravenous zolendronic acid reduced urinary markers of collagen cross linking[11].

Contradictory data from animal studies around the protective effects of bisphosphonates on RRIFs limits our understanding of the pathophysiology and therapeutics of RRIFs. Animal studies using whole mouse radiation have demonstrated an early activation of bone resorption in the 5 days following low dose (2 Gy) of radiotherapy which was reduced by subcutaneous administration of risedronate immediately following irradiation[12]. In contrast, a focal radiation technique in mice (using a small animal radiation research platform or SARRP), arguably a more physiological representative method of irradiation, demonstrated that alendronic acid did not prevent the radiation induced trabecular bone loss but that this was prevented by blocking osteoblast apoptosis with PTH 1-34[13].

The RadBone is the first open-label prospective randomised controlled trial (RCT) to determine the feasibility and acceptability of a musculoskeletal health package (MHP) intervention in women undergoing pelvic radiotherapy for gynaecological malignancies and inform power calculations for a definitive RCT. Moreover, this feasibility trial will also explore potential implications on the incidence of RRIFs, quality of life and other clinical effectiveness and safety outcomes, as well as providing indicative estimates of the intervention's cost-effectiveness.

For peer review only - http://bmjopen.bmj.com/site/about/guidelines.xhtml

Methods and analysis:

Study Design (Figure 1)

Study setting

The planned study is a prospective randomised controlled feasibility trial of eighty patients with gynaecological malignancy (cervical and endometrial) undergoing pelvic radiotherapy at the Christie Hospital NHS Foundation Trust in Manchester, UK (a tertiary referral Oncology centre). The study opened for recruitment in May 2021, and the estimated primary completion date is in November 2022 and study completion date in June 2023.

Eligibility Criteria

Individuals aged over 18 years, with a histologically confirmed endometrial or cervical cancer undergoing potentially curative or adjuvant radiotherapy will be eligible, provided they are able and willing to provide an informed consent to participate.

The exclusion criteria are (i) age less than 18 years or greater than 85 years; (ii) pre-existing bone conditions such as osteoporosis treated with bisphosphonates in the previous 5 years, fibrous dysplasia, osteogenesis imperfecta, or other metabolic bone conditions; (iii) home address outside Greater Manchester; (iv) contraindication or intolerance of Magnetic Resonance scanning.

Interventions

Women undergoing radiotherapy for a gynaecological malignancy will be randomised to an observation (Ob) group and will receive standard assessment and care, following the current local clinical pathway, or an intervention group that will receive a "musculoskeletal health package" (MHP), in addition to standard assessment and care and will be followed for 18 months.

Patients randomised to the MHP arm will receive (i) a physical assessment of musculoskeletal health and a 3-month prehabilitation personalised exercise package as part of the Greater Manchester prehab4cancer program[14], (ii) a fracture risk assessment (FRAX) based on baseline dual-energy x-ray absorptiometry (DXA) bone mineral density (BMD), and (iii) treatment for bone health according to national UK recommendations i.e., standard of

For peer review only - http://bmjopen.bmj.com/site/about/guidelines.xhtml

care for prevention of fragility fractures, by being subdivided into 3 groups (low risk, medium risk and high risk).

Patients with a normal BMD and a FRAX score below the National Osteoporosis Guideline Group (NOGG) recommended treatment line will be considered low risk. Medium risk is defined as osteopenia on the DXA, with FRAX score below the NOGG treatment line. Finally, those with osteopenia and a previous vertebral or hip fracture, or a FRAX score above the NOGG recommended treatment line will be considered high risk.

Low risk patients will be provided with a copy of the Royal Osteoporosis Society (ROS) "Healthy living for strong bones" leaflet. In addition to the leaflet, medium risk patients will receive calcium (1000 mg once daily) and vitamin D (800 IU per day) supplementation. The same interventions will be offered to high-risk patients, who will also undergo secondary osteoporosis screening (blood tests) and will receive oral alendronate 70mg once weekly, in the absence of contraindications. Annual intravenous zolendronic acid infusion will be considered as an alternative where appropriate.

Those randomised to the observation arm will remain blinded to the results of the evaluations until the end of the study unless a fragility fracture or RRIF develops during the study.

Prehabilitation Exercise Programme (Prehab4cancer)

All patients randomised to the MHP arm of the study will be offered a bespoke prehabilitation exercise programme via the Prehab4cancer programme in Greater Manchester. The MHP arm patients will be referred to the Prehab4cancer team via electronic referral immediately following randomisation. Allocated patients will be individually assessed by the Prehab4cancer team according to their usual protocols and assigned an appropriate prehabilitation program. Duration of the programme is 12 weeks from the first assessment and participation will be encouraged, as tolerated. The Prehab4Cancer and recovery programme is community-based, which incorporates exercise (cardiovascular and muscle strengthening/resistance training), nutritional screening, and advice and wellbeing support. Further details of programmes' assessment tools and the stratification of interventions are described by Moore et al[14] and can be found here: www.prehab4cancer.co.uk. The current scope of this protocol is to evaluate feasibility of participants' engagement in this face to face and remote prehabilitation service both pre- and during treatment.

Baseline and Follow-up Evaluation (Figure 2)

For peer review only - http://bmjopen.bmj.com/site/about/guidelines.xhtml

As described in figure 2, baseline evaluations will include a bone health assessment with DXA BMD measurement and completion of a bone health questionnaire. PROMs will also be captured. Finally, fasting serum and plasma blood samples will also be collected.

At 6, 12 and 18 months post radiotherapy all patients will undergo a pelvic MRI assessment for RRIFs, PROMs assessment and fasting blood sampling. During the final visit, at 18 months, all patients will have a DXA BMD scan and physical assessment of their musculoskeletal health. If signs or symptoms compatible with a RRIF are described outside the study visits study participants will be assessed and managed following the current clinical pathways.

Imaging studies

DXA scans of the total hip, femoral neck, L1-L4 spine and TBS assessments will be performed on a single DXA scanner (Hologic Horizon A SN 300792M version 5.6.07 with TBS v.3.0.2 calibrated to the above scanner) at the Christie NHS Foundation Trust as per local protocol. These will be undertaken by two technicians trained in conducting DXA. Images will be reviewed, validated and interpreted by the lead investigator (CEH). The femoral neck BMD (g/cm^2) will be used in conjunction with a standardised DXA questionnaire to complete FRAX calculation.

Pelvic Magnetic Resonance Imaging (MRI) scans will be performed at 6, 12 and 18 months on a 1.5T MRI scanner at the Christie Hospital by trained radiographers in accordance with the study imaging protocol. Four pelvic sequences will be performed per patient (5mm slice thickness, field of view 400mm; coronal T1, coronal STIR (inversion time 150ms), axial T1 and axial STIR (inversion time 165 ms). These will correspond to routine follow-up scans where possible. All bone sequence scans will be dual reported by 2 consultant radiologists who will document the presence of fracture and their confidence in its presence, fracture location, fracture line, bone marrow oedema and other abnormalities.

Biochemical studies

Fasting blood tests will be performed at baseline, weekly during radiotherapy (visits 2 to 10, one day prior to chemotherapy if receiving) and at 6, 12 and 18 months in all patients. Patients allocated to the MHP High risk arm and started on oral bisphosphonate therapy will have an additional bone turnover marker blood test at 3 months to assess bisphosphonate efficacy. All samples will be taken simultaneously with routinely collected clinical blood samples where appropriate.

As part of the MHP intervention arm, blood will be sampled, analysed, and assessed at baseline, 6, 12 and 18 months for the measurement of full blood count, urea and electrolytes, liver function tests, parathyroid hormone, vitamin D, thyroid function test,

For peer review only - http://bmjopen.bmj.com/site/about/guidelines.xhtml

oestradiol, HbA1c, procollagen type 1 amino-terminal propeptide (P1NP), and the beta-C-terminal telopeptide (CTx). Moreover, in the observation arm, serum samples will be collected, processed and stored at -80°C for batch analysis at the end of the study.

Additional fasting blood samples will be collected at all timepoints mentioned for longitudinal analysis of bone turnover markers and for proteomic analysis. These samples will be processed and stored at -80°C, following local standard operating procedures (SOPs), for batch analysis at the end of the study. Bone turnover markers will be evaluated using ELISA techniques and will include CTX, NTX, P1NP, osteocalcin, TRAcP5b and bone ALP.

Sequential Window Acquisition of all Theoretical Mass Spectra (SWATH-MS)

Proteomic analysis will be conducted at the Stoller Biomarker Discovery Centre, following local SOPs[15]. Samples will be analysed by a Data Independent Acquisition method known as SWATH-MS with a micro-flow LC-MS system comprising an Eksigent nanoLC 400 autosampler and an Eksigent nanoLC pump coupled to a SCIEX 6600 Triple-TOF mass spectrometer (68 min run-time). When SWATH maps are generated, the presence and abundance of plasma proteins will be quantified using published plasma reference libraries. Differential expression analysis will be used to identify candidate biomarkers using artificial intelligence approaches. Linear regression will be used to detect correlations with the presence of RRIFs and BMD.

Few longitudinal studies have tracked proteins of interest over the whole course of radiotherapy from pre-treatment baseline through to follow-up. We have undertaken one pilot that shows the potential value of this work[16]. Other studies that have investigated this have demonstrated distinguishing profiles with groups of approximately n=30. Two pre-radiotherapy baseline samples will be used to assess natural variation and comparison with the variance of measurements following radiotherapy and further comparison between the MHP and observation arm (n=40 per group).

Electronic data will be pseudoanonymised (coded) to protect the identity of the participants.

PROMS and Health Utilisation Proforma

PROMs will be collected either as electronic PROMS (using the myChristie, myHealth application) or paper-based PROMS at baseline 6, 12, and 18 months.

The evaluated PROMs will include the adapted pelvic patient-reported outcome version of the common terminology criteria for adverse events (PRO-CTCAE) assessment, the Short

For peer review only - http://bmjopen.bmj.com/site/about/guidelines.xhtml

Musculoskeletal Function Assessment (SMFA) modified for lower limb, the 5-level version of the EuroQol tool (EQ-5D-5L) and a tailored Health Utilisation Proforma.

The CTCAE pelvic questionnaire will include as measures bowel questions scored out of 22, urinary questions out of 19 and sexual questions out of 8, with a total out of 49; a higher score indicates worse quality of life. The adapted SMFA questionnaire includes 39 questions, with a minimum possible score of 39 and maximum of 195; scores are standardised with high scores indicating poor function.

Criteria for discontinuing

Participants may decide to withdraw from the study at any time. Discontinuation of the study participants may occur as a result of investigator decision, safety concerns, and significant non-compliance to the protocol or incorrect enrolment. Reasons for discontinuation will be captured.

As this is a feasibility study, participants may decide to discontinue their participation in certain aspects of the study (for example declining the prehabilitation programme or deciding not to take recommended medications). The participants can continue with the study and the details will be captured in the case report form (CRF).

Outcomes

The primary outcomes for this feasibility study will inform the design and power calculations for a definitive UK multi-centre RCT. These are:

1) Eligibility and screening rate: proportion of patients eligible for the study from patient population. [Assessed at baseline]
2) Recruitment and study group allocation rate: number and proportion of eligible patients recruited, randomised and allocated to appropriate study populations. [Assessed 2 weeks post consent]
3) Intervention fidelity rate: number and proportion of patients completing the elements of the study (assessment visits, prehab exercise programme, prescribed medications, QOL questionnaire). [Assessed at the end of study, at 18 months]
4) Attrition rate: number of patients lost to follow-up. [Assessed at the end of study, at 18 months]
5) Patient and physician acceptability assessed with electronic questionnaires. [Change from baseline assessed at 6,12 and 18 months]
6) Health Economic Analysis: within-trial cost-effectiveness analysis to demonstrate feasibility of health economic data collection and analysis in a multi-centre RCT. [Change from baseline assessed at 6,12 and 18 months]

The secondary outcomes are:

1) Incidence of pelvic Radiotherapy Related Insufficiency Fracture (RRIF). [Assessed at 6, 12 and 18 months post radiotherapy]

For peer review only

2) Longitudinal change in BMD and fracture risk using FRAX. [Assessed at baseline and 18 months]
3) Longitudinal change in biochemical markers of bone turnover. [Change from baseline assessed at 2, 3, 4, 5, 6, 7, 8 and 9 weeks and at 6, 12 and 18 months]
4) Quality of Life assessment: adapted CTCAE pelvic questionnaire and SMFA adapted to lower limbs. [Change from baseline assessed at 6, 12 and 18 months]

Exploratory Endpoints include identification of predictive markers of RRIFs (radiomic, proteomic, BMD) and exploratory measurement of proteomic biomarkers of bone turnover during pelvic radiotherapy.

Sample Size

No formal power calculation has been performed as this is a feasibility study. The study will collect initial data such as measures of location and variability for key outcome measures. It is recognised that in general, 30 patients are required in order to estimate such parameters[17]. For this study a total of 80 patients will be recruited and randomised with equal probability to either the MHP or observation arms (i.e. 40 per group). Assuming attrition rates of 15% per group, at least 30 should remain in each arm. This should be sufficient to assess the feasibility of a larger RCT study and estimate group means, standard deviations and percentages for key outcomes.

Recruitment

80 patients will be recruited over an 18-month period, approximately 4 patients per month. As this is a feasibility study there will be no interim analysis of study results.

Assignment of interventions

Consenting, eligible participants will be randomised to the MHP or observation group using a validated online service; sealedenvelope™ (https://www.sealedenvelope.com). A permuted block (block size:4) randomisation protocol will be utilised with a 1:1 allocation (MHP to observation arm).

Data Collection, management and analysis

Statistical and Health Economic Analysis

As this is a feasibility study, it will not involve hypothesis testing to identify whether the intervention has had an impact. Instead, data analysis will be descriptive, focusing on the percentage of patients in each group developing RRIFs and risk factors for this. Means and a measure of variation will be calculated for each secondary outcome. These data, along with

For peer review only - http://bmjopen.bmj.com/site/about/guidelines.xhtml

estimates of recruitment and attrition rates, will help inform a power calculation for the definitive trial.

A within-trial cost-effectiveness analysis[18] will be undertaken from the perspective of the UK National Health Service (NHS). Cost data for the intervention arm will reflect resource use associated with the musculoskeletal health package and treatment costs for both the control and intervention arm will be taken into account. Resource use will be extracted from patient records and the health care utilisation proforma. Relevant sources (e.g., NHS reference costs) will be used to identify unit costs. Health related quality of life (HRQL) scores will be generated using the EQ-5D-5L at baseline and at each of the three follow-up time points (6, 12, 18 months).

A descriptive analysis of the costs and outcomes data will be completed focusing on: a. whether the EQ-5D-5L and SMFA are able to adequately capture differences in health status before and after implementation of the musculoskeletal health package and across both treatment arms of the study; b. whether the resource-use survey is able to record data necessary to enable a full cost-effectiveness analysis; c. the nature of missing data for the EQ-5D-5L, SMFA and resource-use survey to assess responses, sensitivity, and any patterns within the missing data.

A within-trial cost-effectiveness analysis will be conducted to provide an indicative estimate of cost-effectiveness. Between-arm differences in costs and outcomes will be expressed as an incremental cost-effectiveness ratio (ICERs): the cost per QALY gained from the intervention compared to usual care. ICERs will also be calculated using the SMFA in an additional scenario analysis.

Trial oversight

An internal trial management group will be convened for the study, consisting of the chief investigator, project manager, Clinical Trials administrator, research nurse and a representative of the research and innovation division (R+I) as core members. The group will meet monthly. The study sponsor (Christie Hospital NHS Foundation Trust) will monitor the conduct of the trial.

Patient and Public Involvement

This protocol was developed with the participation of the Christie pelvic radiotherapy user group and supported by the Pelvic Radiation Disease Association (PRDA).

Ethics and Dissemination

For peer review only

For peer review only - http://bmjopen.bmj.com/site/about/guidelines.xhtml

This study has been approved by GM East Research Ethics Committee in November 2020 (REC reference 20/NW/0410) and is registered at clinicaltrials.gov (ClinicalTrials.gov Identifier: NCT04555317). The study opened for recruitment in May 2021. The results of this study will be published in peer reviewed journals, will be presented in national and international conferences, and will be communicated to relevant stakeholders. Moreover, a plain English report will be shared with the study participants, patients' organizations, and media.

Data sharing statement

Consent to share data from this study for future research is voluntary. To ensure compliance with regulatory and governance requirements, approval from the sponsor team is required prior to the release of any data generated by this Christie sponsored study, to a third party. Any requests are to be directed towards the-christie.sponsoredresearch@nhs.net for consideration and must follow all local Policies and review procedures. If a proposal is accepted, then the sponsor will work with the requestor to develop any necessary data transfer plans/agreements.

Authors' contributions: CEH developed the protocol, MRC application and ethics application for the study. RB is the MRC-CARP academic partner to CEH and contributed to the study design and protocol.

KJ, LHB, KH contributed to the development of the gynae-oncology aspects

RK, SO'C, TW contributed to the development of the MRI radiology aspects

ZM, JM contributed to the development of the Prehab4Cancer aspects

ST, JY contributed to the PROMS development

ME contributed to the Health Economic Analysis

ADW, IBJ contributed to the proteomic and data analysis

VCG and CEH prepared the manuscript

All authors: critically reviewed and commented on the manuscript.

Funding statement: This work is partially supported by an MRC-NIHR Clinical Academic Research Partnership) award: grant number MR/T024887/1. Funding is being sought for Health Economic Analysis, QOL and PROMS development and longitudinal proteomic analysis. Equipment used in the Stoller Biomarker Discovery Centre is funded by a donation received from the Stoller Charitable Trust and a research grant awarded by the Medical

For peer review only - http://bmjopen.bmj.com/site/about/guidelines.xhtml

448 Research Council (MR/M008959/1). MRC/EPSRC Molecular Pathology Node provided additional financial support by a grant from the Medical Research Council and Engineering & Physical Sciences Research Council (MR/N00583X/1).

451 **Competing interests statement.** No competing interests

452

For peer review only

For peer review only - http://bmjopen.bmj.com/site/about/guidelines.xhtml

Figures legends

Figure 1: Recruitment, randomisation process and description of the stratified interventions. (#: fracture)

Figure 2: Study flow chart; assessments and outcome time-points.

C+R = Consent and Randomisation, MHP= Musculoskeletal Health Package arm, Ob = observational arm, HR = high risk, CV = clinic visit, EBRT= External Beam Radiotherapy. NHS: National Health System

For peer review only

For peer review only - http://bmjopen.bmj.com/site/about/guidelines.xhtml

Full references

1. Available from: https://www.macmillan.org.uk/_images/cancer-statistics-factsheet_tcm9-260514.pdf [Accessed February 2022]
2. Higham CE, Faithfull S. Bone Health and Pelvic Radiotherapy. Clin Oncol (R Coll Radiol). 2015;27(11):668-678. doi:10.1016/j.clon.2015.07.006
3. Sapienza LG, Salcedo MP, Ning MS, et al. Pelvic Insufficiency Fractures After External Beam Radiation Therapy for Gynecologic Cancers: A Meta-analysis and Meta-regression of 3929 Patients. Int J Radiat Oncol Biol Phys. 2020;106(3):475-484. doi:10.1016/j.ijrobp.2019.09.012
4. Gebauer J, Higham C, Langer T, Denzer C, Brabant G. Long-Term Endocrine and Metabolic Consequences of Cancer Treatment: A Systematic Review. Endocr Rev. 2019;40(3):711-767. doi:10.1210/er.2018-00092
5. van den Blink QU, Garcez K, Henson CC, Davidson SE, Higham CE. Pharmacological interventions for the prevention of insufficiency fractures and avascular necrosis associated with pelvic radiotherapy in adults. Cochrane Database Syst Rev. 2018;4(4):CD010604. Published 2018 Apr 23. doi:10.1002/14651858.CD010604.pub2.
6. Center JR, Nguyen TV, Schneider D, Sambrook PN, Eisman JA. Mortality after all major types of osteoporotic fracture in men and women: an observational study. Lancet. 1999;353(9156):878-882. doi:10.1016/S0140-6736(98)09075-8
7. Taillandier J, Langue F, Alemanni M, Taillandier-Heriche E. Mortality and functional outcomes of pelvic insufficiency fractures in older patients. Joint Bone Spine. 2003;70(4):287-289. doi:10.1016/s1297-319x(03)00015-0
8. Oh D, Huh SJ, Nam H, et al. Pelvic insufficiency fracture after pelvic radiotherapy for cervical cancer: analysis of risk factors. Int J Radiat Oncol Biol Phys. 2008;70(4):1183-1188. doi:10.1016/j.ijrobp.2007.08.005
9. Holch P, Pini S, Henry AM, et al. eRAPID electronic patient self-Reporting of Adverse-events: Patient Information and aDvice: a pilot study protocol in pelvic radiotherapy. Pilot Feasibility Stud. 2018;4:110. Published 2018 Jun 5. doi:10.1186/s40814-018-0304-6
10. Pichon B, Campion L, Delpon G, et al. High-Dose Hypofractionated Radiation Therapy for Noncompressive Vertebral Metastases in Combination With Zoledronate: A Phase 1 Study. Int J Radiat Oncol Biol Phys. 2016;96(4):840-847. doi:10.1016/j.ijrobp.2016.07.027
11. Gierloff M, Reutemann M, Gülses A, Niehoff P, Wiltfang J, Açil Y. Effects of zoledronate on the radiation-induced collagen breakdown: a prospective randomized clinical trial. Clin Transl Oncol. 2015;17(6):454-461. doi:10.1007/s12094-014-1257-8
12. Willey JS, Livingston EW, Robbins ME, et al. Risedronate prevents early radiation-induced osteoporosis in mice at multiple skeletal locations. Bone. 2010;46(1):101-111. doi:10.1016/j.bone.2009.09.002
13. Chandra A, Lin T, Tribble MB, et al. PTH1-34 alleviates radiotherapy-induced local bone loss by improving osteoblast and osteocyte survival. Bone. 2014;67:33-40. doi:10.1016/j.bone.2014.06.030
14. Moore J, Merchant Z, Rowlinson K, et al. Implementing a system-wide cancer prehabilitation programme: The journey of Greater Manchester's 'Prehab4cancer'. Eur J Surg Oncol. 2021;47(3 Pt A):524-532. doi:10.1016/j.ejso.2020.04.042
15. Geary B, Walker MJ, Snow JT, et al. Identification of a Biomarker Panel for Early Detection of Lung Cancer Patients. J Proteome Res. 2019;18(9):3369-3382. doi:10.1021/acs.jproteome.9b00287
16. Walker MJ, Zhou C, Backen A, et al. Discovery and Validation of Predictive Biomarkers of Survival for Non-small Cell Lung Cancer Patients Undergoing Radical Radiotherapy: Two Proteins With Predictive Value. EBioMedicine. 2015;2(8):841-850. Published 2015 Jun 19. doi:10.1016/j.ebiom.2015.06.013
17. Billingham SA, Whitehead AL, Julious SA. An audit of sample sizes for pilot and feasibility trials being undertaken in the United Kingdom registered in the United Kingdom Clinical Research Network database. BMC Med Res Methodol. 2013;13:104. Published 2013 Aug 20. doi:10.1186/1471-2288-13-104
18. Drummond MF, Sculpher MJ, Claxton K, Stoddart GL, Torrance GW. Methods for the Economic Evaluation of Health Care Programmes. 4th ed. Oxford: Oxford University Press, Oxford. <https://books.google.co.uk/books?id=lvWACgAAQBAJ>

For peer review only

For peer review only - http://bmjopen.bmj.com/site/about/guidelines.xhtml

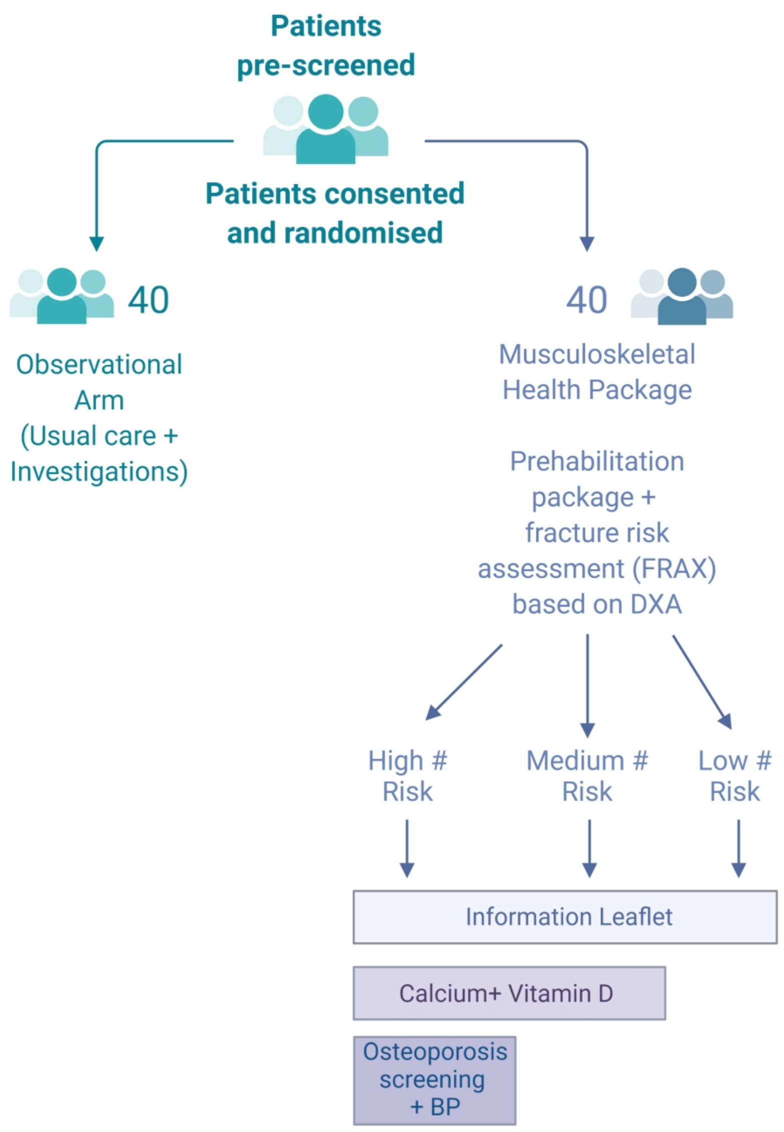

Figure 1: Recruitment, randomisation process and description of the stratified interventions. (#: fracture)

218x310mm (300 x 300 DPI)

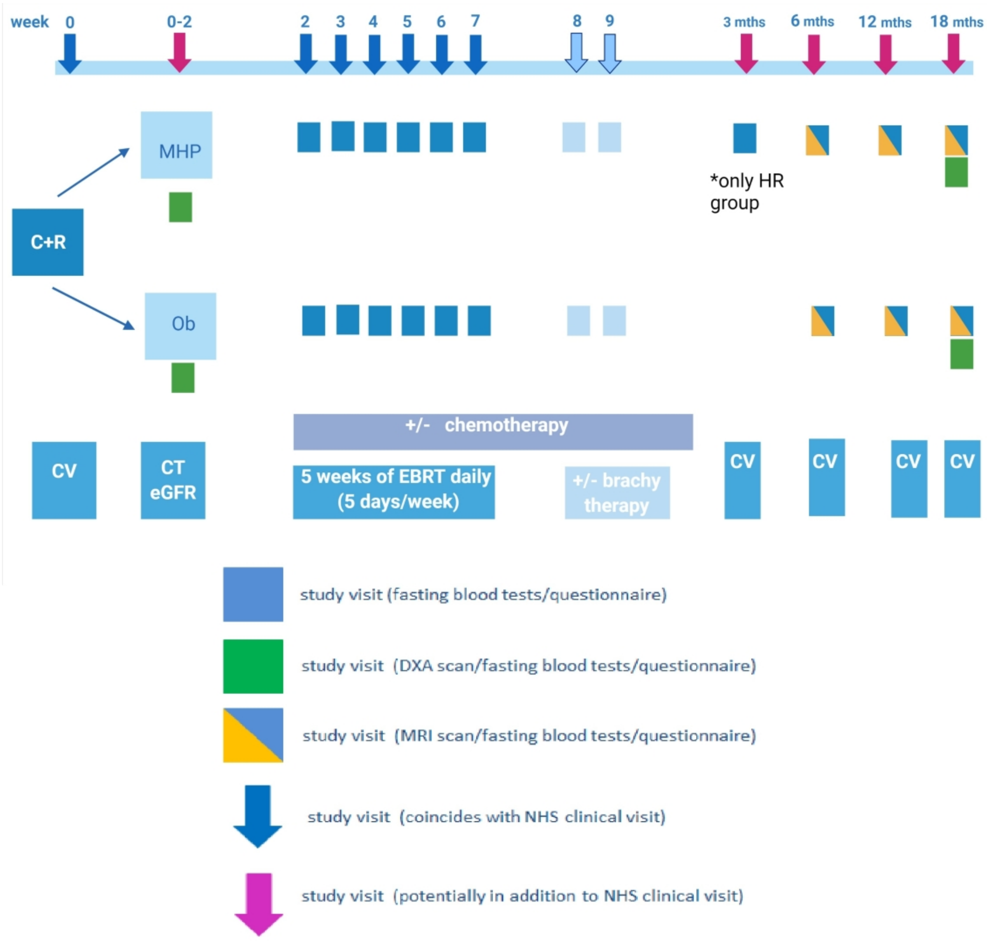

Figure 2: Study flow chart; assessments and outcome time-points.
C+R = Consent and Randomisation, MHP= Musculoskeletal Health Package arm, Ob = observational arm, HR = high risk, CV = clinic visit, EBRT= External Beam Radiotherapy. NHS: National Health System

257x246mm (300 x 300 DPI)

Reporting checklist for protocol of a clinical trial.

Based on the SPIRIT guidelines.

Instructions to authors

Complete this checklist by entering the page numbers from your manuscript where readers will find each of the items listed below.

Your article may not currently address all the items on the checklist. Please modify your text to include the missing information. If you are certain that an item does not apply, please write "n/a" and provide a short explanation.

Upload your completed checklist as an extra file when you submit to a journal.

In your methods section, say that you used the SPIRITreporting guidelines, and cite them as:

Chan A-W, Tetzlaff JM, Gøtzsche PC, Altman DG, Mann H, Berlin J, Dickersin K, Hróbjartsson A, Schulz KF, Parulekar WR, Krleža-Jerić K, Laupacis A, Moher D. SPIRIT 2013 Explanation and Elaboration: Guidance for protocols of clinical trials. BMJ. 2013;346:e7586

		Reporting Item	Page Number
**Administrative information**			
Title	#1	Descriptive title identifying the study design, population, interventions, and, if applicable, trial acronym	1
Trial registration	#2a	Trial identifier and registry name. If not yet registered,	2

For peer review only

		name of intended registry	
Trial registration: data set	#2b	All items from the World Health Organization Trial Registration Data Set	6-14
Protocol version	#3	Date and version identifier	N/A
Funding	#4	Sources and types of financial, material, and other support	14,15
Roles and responsibilities: contributorship	#5a	Names, affiliations, and roles of protocol contributors	14
Roles and responsibilities: sponsor contact information	#5b	Name and contact information for the trial sponsor	13,14
Roles and responsibilities: sponsor and funder	#5c	Role of study sponsor and funders, if any, in study design; collection, management, analysis, and interpretation of data; writing of the report; and the decision to submit the report for publication, including whether they will have ultimate authority over any of these activities	13,14
Roles and responsibilities: committees	#5d	Composition, roles, and responsibilities of the coordinating centre, steering committee, endpoint adjudication committee, data management team, and other individuals or groups overseeing the trial, if applicable (see Item 21a for data monitoring committee)	13,14

Introduction

For peer review only

Background and rationale	#6a	Description of research question and justification for undertaking the trial, including summary of relevant studies (published and unpublished) examining benefits and harms for each intervention	5
Background and rationale: choice of comparators	#6b	Explanation for choice of comparators	5
Objectives	#7	Specific objectives or hypotheses	4,5
Trial design	#8	Description of trial design including type of trial (eg, parallel group, crossover, factorial, single group), allocation ratio, and framework (eg, superiority, equivalence, non-inferiority, exploratory)	5
**Methods: Participants, interventions, and outcomes**			
Study setting	#9	Description of study settings (eg, community clinic, academic hospital) and list of countries where data will be collected. Reference to where list of study sites can be obtained	6
Eligibility criteria	#10	Inclusion and exclusion criteria for participants. If applicable, eligibility criteria for study centres and individuals who will perform the interventions (eg,	7

For peer review only

		surgeons, psychotherapists)	
Interventions: description	#11a	Interventions for each group with sufficient detail to allow replication, including how and when they will be administered	7,8
Interventions: modifications	#11b	Criteria for discontinuing or modifying allocated interventions for a given trial participant (eg, drug dose change in response to harms, participant request, or improving / worsening disease)	11
Interventions: adherance	#11c	Strategies to improve adherence to intervention protocols, and any procedures for monitoring adherence (eg, drug tablet return; laboratory tests)	N/A
Interventions: concomitant care	#11d	Relevant concomitant care and interventions that are permitted or prohibited during the trial	7,8
Outcomes	#12	Primary, secondary, and other outcomes, including the specific measurement variable (eg, systolic blood pressure), analysis metric (eg, change from baseline, final value, time to event), method of aggregation (eg, median, proportion), and time point for each outcome. Explanation of the clinical relevance of chosen efficacy and harm outcomes is strongly recommended	11,12
Participant timeline	#13	Time schedule of enrolment, interventions (including any run-ins and washouts), assessments, and visits for participants. A schematic diagram is highly recommended (see Figure)	8-11

For peer review only

Sample size	#14	Estimated number of participants needed to achieve study objectives and how it was determined, including clinical and statistical assumptions supporting any sample size calculations	12
Recruitment	#15	Strategies for achieving adequate participant enrolment to reach target sample size	12
**Methods: Assignment of interventions (for controlled trials)**			
Allocation: sequence generation	#16a	Method of generating the allocation sequence (eg, computer-generated random numbers), and list of any factors for stratification. To reduce predictability of a random sequence, details of any planned restriction (eg, blocking) should be provided in a separate document that is unavailable to those who enrol participants or assign interventions	12,13
Allocation concealment mechanism	#16b	Mechanism of implementing the allocation sequence (eg, central telephone; sequentially numbered, opaque, sealed envelopes), describing any steps to conceal the sequence until interventions are assigned	12,13
Allocation: implementation	#16c	Who will generate the allocation sequence, who will enrol participants, and who will assign participants to interventions	12,13

For peer review only

Blinding (masking)	#17a	Who will be blinded after assignment to interventions (eg, trial participants, care providers, outcome assessors, data analysts), and how	5
Blinding (masking): emergency unblinding	#17b	If blinded, circumstances under which unblinding is permissible, and procedure for revealing a participant's allocated intervention during the trial	N/a
**Methods: Data collection, management, and analysis**			
Data collection plan	#18a	Plans for assessment and collection of outcome, baseline, and other trial data, including any related processes to promote data quality (eg, duplicate measurements, training of assessors) and a description of study instruments (eg, questionnaires, laboratory tests) along with their reliability and validity, if known. Reference to where data collection forms can be found, if not in the protocol	9-11
Data collection plan: retention	#18b	Plans to promote participant retention and complete follow-up, including list of any outcome data to be collected for participants who discontinue or deviate from intervention protocols	11
Data management	#19	Plans for data entry, coding, security, and storage, including any related processes to promote data quality (eg, double data entry; range checks for data values).	13

For peer review only

		Reference to where details of data management procedures can be found, if not in the protocol	
Statistics: outcomes	#20a	Statistical methods for analysing primary and secondary outcomes. Reference to where other details of the statistical analysis plan can be found, if not in the protocol	13
Statistics: additional analyses	#20b	Methods for any additional analyses (eg, subgroup and adjusted analyses)	13
Statistics: analysis population and missing data	#20c	Definition of analysis population relating to protocol non-adherence (eg, as randomised analysis), and any statistical methods to handle missing data (eg, multiple imputation)	13
**Methods: Monitoring**			
Data monitoring: formal committee	#21a	Composition of data monitoring committee (DMC); summary of its role and reporting structure; statement of whether it is independent from the sponsor and competing interests; and reference to where further details about its charter can be found, if not in the protocol. Alternatively, an explanation of why a DMC is not needed	13,14
Data monitoring: interim analysis	#21b	Description of any interim analyses and stopping guidelines, including who will have access to these interim results and make the final decision to terminate the trial	12
Harms	#22	Plans for collecting, assessing, reporting, and managing solicited and spontaneously reported adverse events and other unintended effects of trial interventions or trial	11

For peer review only

		conduct	
Auditing	#23	Frequency and procedures for auditing trial conduct, if any, and whether the process will be independent from investigators and the sponsor	14
**Ethics and dissemination**			
Research ethics approval	#24	Plans for seeking research ethics committee / institutional review board (REC / IRB) approval	2
Protocol amendments	#25	Plans for communicating important protocol modifications (eg, changes to eligibility criteria, outcomes, analyses) to relevant parties (eg, investigators, REC / IRBs, trial participants, trial registries, journals, regulators)	13,14
Consent or assent	#26a	Who will obtain informed consent or assent from potential trial participants or authorised surrogates, and how (see Item 32)	7
Consent or assent: ancillary studies	#26b	Additional consent provisions for collection and use of participant data and biological specimens in ancillary studies, if applicable	N/A
Confidentiality	#27	How personal information about potential and enrolled participants will be collected, shared, and maintained in order to protect confidentiality before, during, and after the trial	13
Declaration of	#28	Financial and other competing interests for principal	14,15

For peer review only

interests		investigators for the overall trial and each study site	
Data access	#29	Statement of who will have access to the final trial dataset, and disclosure of contractual agreements that limit such access for investigators	TBC
Ancillary and post trial care	#30	Provisions, if any, for ancillary and post-trial care, and for compensation to those who suffer harm from trial participation	N/A
Dissemination policy: trial results	#31a	Plans for investigators and sponsor to communicate trial results to participants, healthcare professionals, the public, and other relevant groups (eg, via publication, reporting in results databases, or other data sharing arrangements), including any publication restrictions	14
Dissemination policy: authorship	#31b	Authorship eligibility guidelines and any intended use of professional writers	14
Dissemination policy: reproducible research	#31c	Plans, if any, for granting public access to the full protocol, participant-level dataset, and statistical code	14
**Appendices**			
Informed consent materials	#32	Model consent form and other related documentation given to participants and authorised surrogates	TBC
Biological specimens	#33	Plans for collection, laboratory evaluation, and storage of biological specimens for genetic or molecular analysis in the current trial and for future use in ancillary studies, if applicable	N/A

For peer review only

None The SPIRIT Explanation and Elaboration paper is distributed under the terms of the Creative Commons Attribution License CC-BY-NC. This checklist can be completed online using https://www.goodreports.org/, a tool made by the EQUATOR Network in collaboration with Penelope.ai

For peer review only
